# Morphology and Histology of the Orbital Region and Eye of the Asiatic Black Bear (*Ursus thibetanus*)—Similarities and Differences within the Caniformia Suborder

**DOI:** 10.3390/ani12070801

**Published:** 2022-03-22

**Authors:** Wojciech Paszta, Karolina Goździewska-Harłajczuk, Joanna Klećkowska-Nawrot

**Affiliations:** 1 Wroclaw Zoological Garden, Wróblewskiego 1/5, 51-618 Wrocław, Poland; 2 Department of Biostructure and Animal Physiology, Faculty of Veterinary Medicine, Wrocław University of Environmental and Life Sciences, Kożuchowska 1, 51-631 Wrocław, Poland; joanna.kleckowska-nawrot@upwr.edu.pl

**Keywords:** anatomy, histology, eye area, eyelids, lacrimal gland, Asiatic black bear

## Abstract

**Simple Summary:**

The aim of this study was the anatomical description of the orbital region, eye tunics, upper and lower eyelids, superficial gland of the third eyelid with the third eyelid, and lacrimal gland of the Asiatic black bear. The eyeball morphometry, the orbital region analysis, macroscopic, morphometric and histological analysis of the eye tunics and anatomical analysis of the accessory organs of the eye with histochemical methods were used. The anatomical structures within the orbital region of Asiatic black bear were typical of only the family Ursidae and were similar to the family Canidae. The macroscopic and histological structure of the eye and eyelids was comparable to the structure of the eye and eyelids in other representatives of the Ursidae family, although features typical of only Canidae or terrestrial Mustelidae and Pinnipedia were also observed. The Bowman’s membrane of the cornea was absent, similar to all domestic dogs and some wild dogs. The pupil was similar to other bear species subfamilies, Tremarctinae and Ursinae, and also to domestic and wild dogs. The lens was biconvex round, similar to the Canidae. The retina was composed similarly to the diurnal terrestrial carnivores. In both eyelids were observed very well-developed tarsal glands, ciliary glands and sebaceous glands. The third eyelid was T-shaped and composed of the hyaline tissue and contained conjunctival-associated lymphoid tissue (CALT), which was also the case in Canidae. The obtained results indicate that the features of the anatomy of the eye and orbital region in the Asiatic black bear are also typical of the Ursidae family.

**Abstract:**

In this study, we present first data concerning the morphological observations of the orbital region, eye tunics, upper and lower eyelids, superficial gland of the third eyelid with the third eyelid, and lacrimal gland in captive adult male Asiatic black bear. The following research methods were used in the work: the eyeball morphometry, the orbital region description, macroscopic description, morphometric and histological analysis of the eye tunics and selected the accessory organs of the eye (Fontana–Masson, hematoxylin & eosin (H&E), Methyl-green-pyronin Y (MGP Y), Movat pentachrome, and picro-Mallory trichrome) as well as histochemical examination (PAS, AB pH 1.0, AB pH 2.5, AB pH 2.5/PAS and HDI). The eyeball of the Asiatic black bear was a spherical shape, while the periorbita was funnel/conical-shaped and the eye socket was of the open type. The cornea was absent of the Bowman’s membrane similar to all domestic dogs and some wild dogs. There were palisades of Vogt in the corneal limbus epithelium similar to the Canidae. Degenerative choroidal tapetum lucidum similar to ranch mink (Mustelidae) has been found. The pupil was big and round in shape. The ciliary muscle, dilatator and sphincter muscle were well developed, similar to the pinnipeds. The lens was biconvex round, similar to the Canidae. The retina was composed similarly to the diurnal terrestrial carnivores. In both eyelids were observed very well-developed tarsal glands, ciliary glands and sebaceous glands. The orbital zone in the eyelids was characterized by lymphoid follicles, diffuse lymphocytes and specialized high endothelial venules. In the anterior palpebral margin of the upper eyelid, soft and short eyelashes were observed, while in the lower eyelids they were absent. The third eyelid was T-shaped and composed of the hyaline tissue, and it contained CALT, similar to that in Canidae. The superficial gland of the third eyelid was a multilobar alveolar branched complex with seromucous nature, while the lacrimal gland was also a multilobar acinar branched complex gland, but producing a mucous–serous secretion. The results of our research indicate that the features of the anatomy of the eye and orbital region in Asiatic black bear are also typical of the Ursidae family. Moreover, a detailed analysis of the morphological eye region may be useful in comparative studies and veterinary diagnostics in this bear species.

## 1. Introduction

The Himalayan bear (*Ursus thibetanus*), also known by another name as the moon bear or the ring bear, was previously known as *Selenarctos thibetanus*. It occurs in seven subspecies [1,2], and its range includes a large part of southern Asia, from Pakistan on the west through eastern Siberia and into North and South Korea and Japan [3,4]. The upper limit of the observation of the Himalayan bear is 4300 m above sea level in summer, while in winter, bears can descend to a height of about 1500 m above sea level, preferring deciduous and mixed forests [3]. A characteristic feature of this genre is solid black often paler on the muzzle and face fur and usually with a characteristic crescent-shaped or V-shaped patch on the chest [3,5].

The *Ursus thibetanus* bears are active during the day, while in areas where they have excessive contact with humans, they prefer a night lifestyle. They are the largest arboreal mammals, spending about 50% of their time on them [6]. They build their lairs from branches at a height of about 4 m [6]. In the cold northern parts of their range, these animals may hibernate between November and April, while in warmer regions, the hibernation period is shorter, or they migrate to warmer regions. Natural predators that pose a threat to both adult and young Asian black bears are Siberian tigers (*Panthera tigris altaica*), brown bear (*Ursus arctos*), leopard (*Panthera pardus*), and sometimes wolves (*Canis lupus*) and Eurasian lynx (*Lynx lynx*) [7]. 

These animals use all possible food sources, including agricultural produce and livestock, and are therefore often killed at the hands of farmers [3,8,9]. Another very serious threat to this species, despite legal protection, is hunting to obtain fur, meat, trophies (paws), and criminal trapping for bile, highly valued in Chinese folk medicine [3,10].

According to Brown, 2009 [3], and Hunter, 2011 [11], bears are more naturally diurnal than nocturnal, where the greater period of activity falls on daylight hours, of course, there are also differences between species and individual animals, where diurnal activities dominate. The increased nocturnal activity of some bears is also an adaptive trait due to some geographic and seasonal factors (human presence/increased human activity in bear territory) but also an impact (development, recreation and hunting) increase [3,11]. The increased period of bear activity also depends on food requirements but is also affected in some bears by temperature, weather and lunar phases [3]. Brown, 2009 [3], suggests that any bear can be active at any time.

There are currently 68 institutions (zoos) housing Asian black bears, in which there are 257 individuals on 3 continents (108 males, 146 females, 3 individuals of undetermined sex). In Europe, there are 61 individuals in 29 zoos (24 males, 37 females), while in Poland, only 4 individuals are found in 3 zoos—Wroclaw (2 females), Toruń (1 female) and Chorzów (1 female) (date from 16 October 2021) (Species 360.org).

The first descriptions of eyesight in the bear appeared at the beginning of the 20th century [12,13,14,15]. Kuckuk, 1937 [15], states that the brown bear *Ursus arctos* has quite adequate visual capacities. Kuckuk, 1937 [15], found that he could be recognized by the two young bears being studied in a crowd of strangers at a distance of 15 m. Couturier, 1954 [16], remarks that brown bears are capable of discriminating bright colors but not the more tonal pastels. According to Bacon, 1973 [14], and Shepard and Sanders, 1985 [17], the eyesight of bears has long been thought to be generally poor, which could probably be related to well-developed olfactory and vestibulocochlear organs [18]. However, new research by scientists has shown that their vision is reasonably good, though there is still much to be learned of the visual capabilities of each bear species. The bear’s eyes are characterized by various shades of brown (polar bear *Ursus maritimus* eyes appear black but are a golden brown; sometimes they are greenish-yellow in the dark), being blue at birth (gradually become brown); large polar bears are an exception; the pupils of bears are round, except for those of the giant panda *Ailuropoda melanoleuca*, which are vertical slits, possibly for improved night vision, and are widely spaced and face forward [3]. Research by Kelling et al., 2006 [18], describing color vision in the giant panda showed that these bears were able to discriminate shades of green, red, and blue stimuli from numerous shades of gray. The authors also noted that “brightness was eliminated as a cue, these discriminations can be attributed to color vision, thus providing experimental evidence that the giant panda’s visual capabilities, including color vision” are comparable to those not only of other bears but also other carnivores that are not strictly nocturnal animals [19,20,21]. In addition, bear sight is characterized by providing excellent night vision, good peripheral vision, good depth perception (at closer distances), observing moving objects better than stationary objects and being binocular [3]. Particularly noteworthy are the most specialized eyes of the polar bears, where the presence of a third eyelid allows for good underwater vision, but also cleans and protects the eyes from the severe arctic glare [3,22]. In addition, the polar bear’s eyes adapt to a wide range of light conditions, including darkness for hunting at night or during the dark winter [3]. Recent research on the American black bear by Heyward et al., 2020 [23], reports that these bears are predicted to have a dichromatic vision with high acuity indicated by the presence of a dorsal temporal located area centralis. 

The literature describing the correct structure of the skull while taking into account the anatomical description of the orbital region, the eyeball and the accessory organs of the eye at the macro and microscopic level in the family Ursidae (American black bear *Ursus americanus* [23,24]; Asiatic black bear *Ursus thibetanus* [25]; North American brown bear or Grizzly bear *Ursus arctos horribilis* [26,27]; brown bear *Ursus arctos* [28,29,30,31], sloth bear *Melursus ursinus* [25,32,33,34]; Sun Bear *Ursus malayanus* [25]; polar bear *Ursus maritimus* [27]) are very limited. On the other hand, studies on clinical case descriptions of diseases of the eyeball and accessory organs of the eye in representatives of this family are much more frequent [34,35,36,37,38,39,40,41,42,43,44,45,46,47,48,49,50,51,52]. A review of the literature describing the morphology of the eye socket, eye tunics, eyelids and orbital glands in suborder Caniformia is presented in Table S1.

The aim of this study was the investigation of the anatomical, histological and histochemical aspects of the Asiatic black bear orbital region, eyeball and selected accessory organs of the eye. Additionally, our research aimed to compare macro- and micromorphological findings of the selected ocular structures with those of other species of a bear falling into the rocks of the Ursidae family and other representatives of the Caniformia suborder, to reveal similarities and differences among these animals. This work can also be a valuable source of information for veterinary ophthalmologists working in national parks and zoos for diagnostic tests and surgical procedures performed within the eye in this species of a representative of the Ursidae family.

## 2. Materials and Methods

### 2.1. Collection of Specimen and Conservation Status

**Figure 1:**
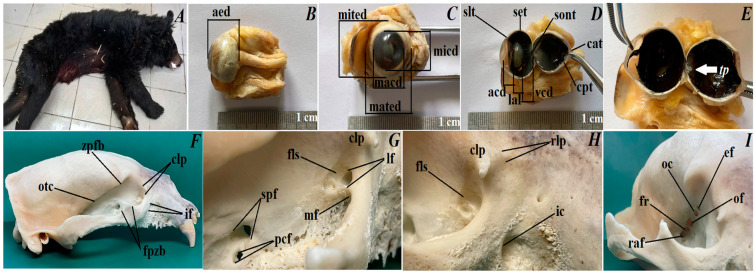
The macrograph of the adult male *Ursus thibetanus* (**A**); dimensions of the eyeball (**B**–**D**); *tapetum lucidum* (**E**); skull (**F**); orbital anatomy (**F**–**I**). acd—aqueous chamber depth, aed—axial eye diameter, cat—corneal (axial) thickness, clp—caudal lacrimal process, cpt—corneal (peripheral) thickness, ead—axial eye diameter, ef—ethmoid foramen, fls—fossa for the lacrimal sac, fpzb—frontal process of the zygomatic bone, fr—foramen rotundum, ic—infraorbital canal, if—infraorbital foramen, lal—lens axial length, lf—lacrimal foramen, macd—maximum corneal diameter, mated—maximum transverse eye diameter, mf—maxillary foramen, micd—clpminimum corneal diameter, mited—minimum transverse eye diameter, oc—optic canal, of—orbital fissure, otc—orbitotemporal crest, pcf—palatine caudal foramen, raf—rostral alar foramen, rlp—rostral lacrimal process, set—scleral (equator) thickness, slt—scleral (limbus) thickness, sont—scleral (optic nerve) thickness, spf—sphenopalatine foramen, *tp—tapetum lucidum* (white arrow), vcd—vitreous chamber depth, zpfb—zygomatic process of the frontal bone.

The research material was taken from one male of Asiatic black bear [53] (aged 27 years, 4 months, and 10 days) coming from Wroclaw Zoological Garden (Poland) (2018) (Figure 1A). The test samples were obtained post-mortem after the animal was euthanized at the Wroclaw Zoological Garden due to unhealed wounds associated with a non-wolf defect in the maxillary alveolar arch.

### 2.2. Ethical Statement

According to Polish and European law, studies on tissues obtained post-mortem do not require approval of the Ethics Committee (2010/63/EU Directive of the European Parliament and of the Council of 22 September 2010 on the protection of animals used for scientific purposes) and The Journal of Laws of the Republic of Poland, the Act of 15 January 2015, on the protection of animals used for scientific or educational purposes. Personal permits issued by the District Veterinary Doctor in Wrocław (Poland) (No. PIW Wroc. UT-45/5/16, No. PIW Wroc. UT-45/6/16, No. PIW Wroc. UT-45/8/16).

### 2.3. Gross Macroscopy Analysis

The following were collected for the research: eyeballs (*n* = 2), upper eyelids (*n* = 2), lower eyelids (*n* = 2), superficial gland of the third eyelid (*n* = 2), third eyelid (*n* = 2) and lacrimal gland (*n* = 2). Muscle of eyeball, orbital fat body, and fascial sheath of eyeball have been partially removed for microscopic examination from eyeballs. *Nomina Anatomica Veterinaria* (2017) [54] and *Nomina Histologica Veterinaria* (2017) [55] were used to describe the anatomical and histological description of the examined structures (eye tunics, eyelids and orbital glands). The eyeballs were measured according to the methods described by Hermanson et al., 2020 [56] (Figure 1B–E):

- Axial eye diameter: from the corneal vertex to the root of the optic nerve;
- Maximum transverse (equatorial) eye diameter;
- Minimum transverse (equatorial) eye diameter;
- Maximum and minimum corneal diameter;
- Corneal axial thickness and corneal peripheral thickness;
- Lens axial length;
- Aqueous chamber depth;
- Vitreous chamber depth;
- Tapetum lucidum length;
- Tapetum lucidum thickness.

Morphometric measurements of eyeballs (see points a–j), eyelids and orbital glands (length, width, thickness) were measured using a digital caliper (Stainless Hardened, Farnell, Poland). Due to the analysis of only these Asiatic black bears, no statistical tests were used in the following work. The values of six randomly measurements of the whole eyeballs, eyelids and orbital glands were recorded. The obtained measurements were analyzed statistically: mean and standard deviation (S.D.). The anatomy of the orbital region of the Asiatic black bear was analyzed based on the anatomical description of the orbit by Nickel et al., 2004 [57], and *Nomina Anatomica Veterinaria* (2017) [54].

### 2.4. Light Microscopic Studies 

Immediately after the death of the animal, the eyeballs with selected additional organs of the eye were subjected to preparation, morphometry, and then the collected samples were placed in 4% buffered formaldehyde for at least 72 h and then rinsed in running water for 24 h. Then, the samples were processed in a vacuum tissue processor—ETP (RVG3, Intelsint, Pantigliate, Italy) and embedded in paraffin. The specimens were cut using a Slide 2003 (Pfm A.g., Cologne, Germany) sliding microtome into 4 µm sections. The following histological stains were performed:

Fontana–Masson: for visualization of melanin (black color);

Mayer’s hematoxylin and eosin: for general histological description;

Picro-Mallory trichrome: for detection of different components of connective tissue (elastic fibers—pale pink to yellow color, collagen fibres—dark blue color, mucus—shades of blue color);

Methyl-green-pyronin Y: for demonstration of plasma cells (cytoplasm of plasma cells—pink to red color, nucleus—dark pink to red color);

Movat pentachrome (modified Russell Movat): for demonstration of collagen and reticular fibers (yellow color), elastic fibers (black to blue/black color), muscle (red color), mucin (bright blue color) and fibrin (bright red color).

The slides obtained were then observed using the Zeiss Axio Scope A1 light microscope (Carl Zeiss, Jena, Germany) and rated a scoring system based on a standard protocol previously described [58,59,60]. The histological measurements of selected eyeball structures were performed with the Axio Vision Rel. 4.8 Software (Carl Zeiss. Jena, Germany). Histochemical examination of analyzed structures (cornea—only PAS stain, eyelids and orbital glands) was performed according to on a standard protocol previously described to Spicer and Henson, 1967 [61], where (−) indicated a negative reaction; (−/+) and (+) a weakly positive reaction; (+/++) and (++) a moderate positive reaction and (+++) a strong positive reaction. The following histochemical stains were performed:

Periodic acid-Schiff (PAS): for identification of glycans, glycoconjugates, neutral or weakly acid glycoproteins (magenta color); 

Alcian blue pH 1.0 (AB pH 1.0): for visualization of strongly sulfated mucosubstances (blue color);

Alcian blue pH 2.5 (AB pH 2.5): for demonstration of acid mucopolysaccharides (dark blue color);

Alcian blue pH 2.5 PAS (AB pH 2.5/PAS): for identification of acidic sulfated mucosubstances and sialomucins (blue color) and neutral mucins (magenta color);

Hale’s dialyzed iron (HDI) for visualization of carboxylated and sulfated mucopolysaccharides and glycoproteins (deep blue color). 

All histochemical stains were performed based on the following analyses: Bancroft and Gamble, 2008; Carson, 1990; Munakata et al., 1985; Sheehan and Hrapchak 1980 [62,63,64,65].

## 3. Results

### 3.1. The Eyeball Morphometry and Orbital Region Description 

The eyeball. The eyeball in the Asiatic black bear has a spherical shape. The axial eye diameter was 16.7 ± 0.4 mm, approximately. The maximum transverse eye diameter was 17.127 ± 0.09 mm, and the minimum transverse eye diameter was 18.143 ± 0.08 mm, approximately. The maximum corneal diameter was 12.404 ± 0.2 mm, and the minimum corneal diameter was 11.56 ± 0.2 mm, approximately. The scleral (limbus) thickness was 1.315 ± 0.09 mm approximately, while the scleral (equator) thickness was 0.903 ± 0.3 mm, approximately and the scleral (optic nerve) thickness was 0.61 ± 0.09 mm, approximately. The corneal axial thickness was 0.851 ± 0.04 mm, and the corneal peripheral thickness was 0.835 ± 0.05 mm, approximately. The aqueous chamber depth was 2.013 ± 0.6 mm, while the vitreous chamber depth was 5.578 ± 0.1 mm and the lens axial length was 6.305 ± 0.08 mm, approximately. Macroscopic examinations revealed the presence of a very faintly marked tapetum lucidum which resembled a crescent moon with a dark brown color, being slightly opalescent. The length of tapetum lucidum was 8.063 ± 0.05 mm, while the width of tapetum lucidum in the narrower part was 3.573 ± 0.08 mm, and in the wider part was 6.719 ± 0.1 mm, approximately.

The orbital region. The periorbita in the Asiatic black bear was funnel/conical-shaped and the eye socket was of the open type. The orbital ring was filled with an orbital ligament connecting the very high frontal process of the zygomatic bone with clearly marked the zygomatic process of the frontal bone. The bones included in the orbital region were: an orbital part of the frontal bone, a small and narrow facial and orbital surface of the lacrimal bone, a large sphenoidal process of the palatine bone and a large pterygoid process of basisphenoid bone. The orbital surface of a body of maxilla was marked and it was in contact with the pterygopalatine surface, where a large maxillary foramen was located intranasally, which passed through a very short infraorbital canal (about 1.3–1.4 cm) into the infraorbital foramen located at the height of the 3rd tooth cheek. There were two smaller holes on the perpendicular plate of palatine bone: sphenopalatine foramen, and palatine caudal foramen directly behind it. On the facial surface of the lacrimal bone in the nasal direction, there was a small prominence—rostral lacrimal process—and on the orbital edge of the lacrimal bone, there was a large millet grain size—caudal lacrimal process. The supraorbital foramen, trochlear fovea for the superior oblique muscle and fossa of ventral oblique muscle were absent in the Asiatic black bear. The fossa for the lacrimal sac, which lead through a single lacrimal foramen to the lacrimal canal, was present on the orbital surface of the lacrimal bone. The temporal surface of the frontal bone had a sharp and very clearly exposed orbitotemporal crest. Directly below the above-mentioned crest was a single medium-sized ethmoid foramen for the n. Ethmoidalis. About 1.3–1.4 cm from the ethmoid foramen was the optic canal for the optic nerve, which ran within the wings of the presphenoidal bone. Then, about 2.1–2.2 cm from the optic canal, above the pterygoid crest, was located a large orbital fissure, and directly below was also a large foramen rotundum, which was present on the wings of the basisphenoid bone. The orbital fissure and foramen rotundum form the route for n. Ophthalmicus, n. Oculomotorius, n. Trochlearis, n. Abducens and n. Maxillaris. Downstream of the foramen rotundum was the rostral alar foramen, which led via the alar (pterygoid) canal (approximately 1.2–1.3 cm long) to the caudal alar foramen (Figure 1F–I).

### 3.2. Macroscopic Observations of the Eyelids and Orbital Glands

**Figure 2:**
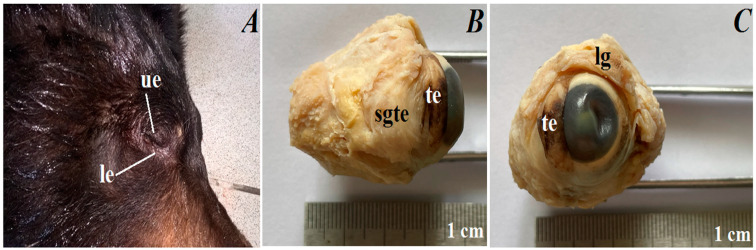
Gross macroscopic image of the *Ursus thibetanus* eyelids (**A**,**B**) and orbital glands (**B**,**C**). le—lower eyelid, lg—lacrimal gland, sgte—superficial gland of third eyelid, te—third eyelid, ue—upper eyelid.

The upper and lower eyelids. In the anterior palpebral margin of the upper eyelid, soft and short eyelashes were observed, while in lower eyelids they were absent (Figure 2A). The palpebral conjunctiva was very pigmented (had black-brown color). The upper eyelid was approximately 22.555 ± 0.8 mm long × 9.166 ± 0.1 mm wide × 5.34 ± 0.1 mm thick, and the lower eyelid was approximately 22.555 ± 0.8 mm long × 8.784 ± 0.3 mm wide × 5.661 ± 0.3 mm thick.

The superficial gland of the third eyelid. The superficial gland of the third eyelid was small (approximately 8.241 ± 0.2 mm long × 10.44 ± 0.4 mm wide × 4.815 ± 0.2 mm thick) (was wider than longer), oval in shape and had a light pink color (Figure 2B). These glands were located in the medial corner of the eye, between medial straight and ventral straight muscles, and were partially covered by the ventral oblique muscles.

The third eyelid. The marginal part of the third eyelids was very pigmented (brown-black color) and was thick. The third eyelid was T-shaped and consisted of an upper and lower branch (the length of the upper and lower branch together was approximately 14.003 ± 0.5 mm) and a crossbar (length was approximately 8.347 ± 0.3 mm) (Figure 2B). The third eyelid was located in the medial canthus of the eye.

The lacrimal gland. The lacrimal gland was very small (approximately 8.819 ± 0.2 mm long × 5.2 ± 0.1 mm wide at its widest point × 3.596 ± 0.2 mm thick), triangular in shape and was light pink in color (Figure 2C). It was located in the dorsal-lateral corner of the eye, between the dorsal straight and lateral straight muscles.

### 3.3. Histological Observations of the Eye Tunics, Eyelids and Orbital Glands

**Figure 3:**
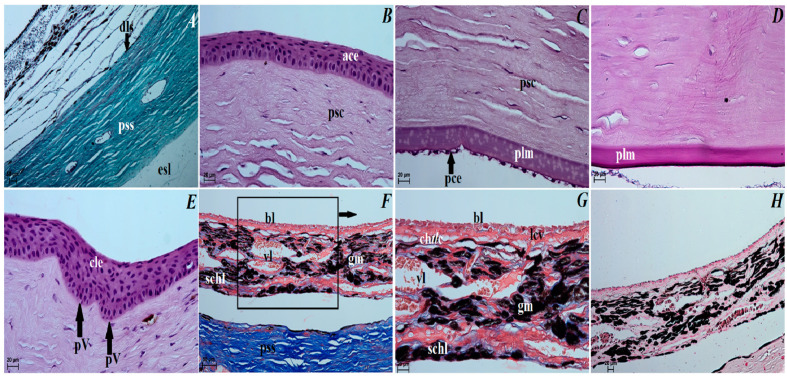
Photomicrograph of the *Ursus thibetanus* eyeball tunics. Sclera (**A**), Movat pentachrome stain; Cornea (**B**,**C**), H&E; Cornea (**D**) and the posterior limiting membrane (Descemet’s membrane) with strongly (+++) positive PAS reaction; Corneal limbus epithelium (**E**) with palisades of Vogt, H&E; Choroid (**F**,**G**), Movat pentachrome stain; Choroid (**H**) with black in color granules of melanin; Fontana–Masson stain. ace—anterior corneal epithelium, bl—basal lamine, cle—corneal limbus epithelium, ch*tl*c—choroidal *tapetum lucidum* cellulosum, dls—dark lamina of sclera, esl—episcleral lamina, gm—granules of melanin, lcv—lamina of capillary vessels, pce—posterior corneal epithelium, plm—posterior limiting membrane, psc—proper substance of cornea, pss—proper substance of sclera, pV—palisades of Vogt, schl—suprachoroid layer, vl—vascular layer. Scale bars: (**A**,**F**) = 50 µm; (**B**–**E**,**G**,**H**) = 20 µm.

**Figure 4:**
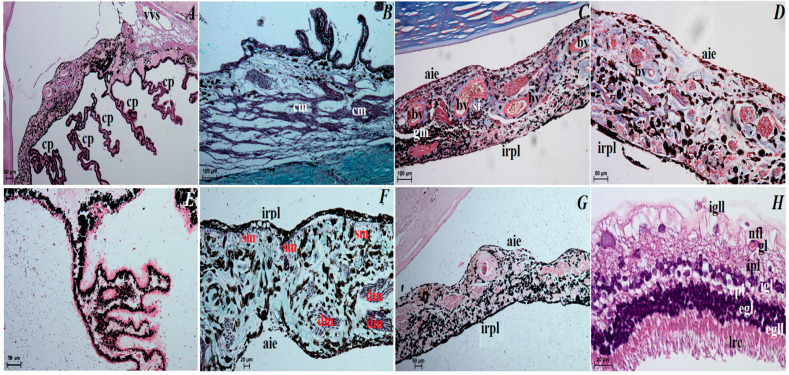
Photomicrograph of the *Ursus thibetanus* eyeball tunics. Ciliary body (**A**) with ciliary processes, H&E staining; Ciliary body (**B**) with ciliary muscle, Movat pentachrome stain; Iris (**C**,**D**), picro-Mallory trichrome stain; Iris (**E**) with sphincter and dilatator muscles, Movat pentachrome stain; (**F**) Ciliary processes with granules of melanin, Fontana–Masson stain; Iris (**G**) with granules of melanin, Fontana–Masson stain; Retina (**H**), H&E. aie—anterior iris epithelium, bv—blood vessels, cm—ciliary muscle, cp—capillary processes, dm—dilatator muscle, egl—external granular layer, egll—external glial limiting layer, epl—external plexiform layer, gl—ganglionic layer, gm—granules of melanin, igl—inner granular layer, igll—inner glial limiting layer, ipl—inner plexiform layer, irpl—iris pigmentosum layer, lrc—layer of rods and cones, nfl—nervous fiber layer, psi—posterior surface of iris, si—stroma of iris, sm—sphincter muscle, vvs—venous sinus of sclera. Scale bars: (**B**,**C**) = 100 µm; (**A**,**G**,**E**) = 50 µm; (**F**,**H**) = 20 µm.

The eye tunics. The sclera consisted of the following three layers: the episcleral lamina, which consisted of loose fibrous connective tissue containing collagen fibers and elastic and, to a lesser extent, reticulated fibers, as well as connective tissue cells (fibrocytes, fibroblasts, histiocytes and mast cells); the middle layer, defined as the proper substance of sclera consisted of collagen fibers and weakly marked elastic fibers that formed distinct lamellae, arranged to the eyeball surface, sparse granules of melanin clusters, sparse blood vessels; the thin dark lamina of sclera layer, being in close contact with the suprachoroid layer and composed of fibroblasts, few blood vessels and melanin granules (Figure 3A). The cornea consisted of only 4 layers as no anterior limiting membrane (Bowman’s membrane) was found: the anterior corneal epithelium—noncornified stratified squamous epithelium that consisted of five–six layers of cells arranged in peripheral part and six–seven layers of cells in the axial part (basal epithelial layer was 1–3 layers of large cylindrical cells); the proper substance of cornea composed of dense fibrous connective tissue made of collagen fibers with a layered parallel arrangement and numerous flattened corneocytes; the posterior limiting membrane (Descemet’s membrane) made of regularly arranged collagen fibers characterized by PAS strongly (+++) positive reaction; and the posterior corneal epithelium constituting a single-layer squamous epithelium with strongly spindle-shaped cells (Figure 3B–D). The anterior corneal epithelium thickness in the peripheral part was 52,628 ± 1.4 µm, and in the axial part was 45,971 ± 2.6 µm; the proper substance of cornea thickness was 727.067 ± 6.3 µm and the posterior limiting membrane thickness was 22.79 ± 1.4 µm. The corneal limbus was situated on the border between the sclera and cornea. Palisades of Vogt were present within the corneal limbus epithelium. The limbal epithelium was composed of 13 layers of epithelial cells: 3 layers of flattened superficial cells with a squamous nucleus, 6 layers of intermediate wing cells with an oval nucleus, and 4 layers of basal cells with a round nucleus. In all of the cell layers, a few scattered granules of melanin were present (Figure 3E). The sclera venous sinus was lined with a flat endothelium. In the corner of the anterior chamber of the eyeball, there was a densely woven mesh of delicate collagen trabeculae sent out through the endothelium. No granules of melanin were observed around the venous sclera sinus (Figure 4A). The choroid consisted of the suprachoroid layer, which is composed of the following: dense fibrous connective tissue with very numerous melanocytes; the vascular layer composed of ciliary arteries and large diameter vortex veins between which there was loose fibrous connective tissue with dominant elastic and collagen fibers and visible granules of melanin; the choroidal tapetum lucidum cellulosum, which is composed of loosely packed degenerative cells with a diameter between 18.81 ± 10.4 µm and 40.007 ± 9.1 µm arranged in 2–3 layers, surrounded by collagen fibers and numerous granules of melanin (the wallpaper cells characterized a nonregular internal arrangement of membranes); the lamina of capillary vessels and the basal layer (Bruch’s membrane) consisted with endothelial cells of the capillary lamina, collagen and elastic fibers; the basal membrane of the retinal non-pigmented layer (Figure 3F–H). The choroid thickness was 185.00 ± 14.08 µm. The ciliary body was big, and it had a round shape. The ciliary processes with a length of 455.84 ± 51.09 µm were covered with a cylindrical bilayer epithelium in the stroma of which numerous blood vessels were observed. The outer layer had no dye, while the inner layer was rich in granules of melanin (Figure 4E). The ciliary body included the very highly evolved ciliary muscle (Figure 4A,B). The iris had a black-brown color and it consisted of the following: the anterior iris epithelium, which was composed of a single-layer squamous epithelium; the outer limiting layer was composed of collagen fibers with fibrocytes and granules of melanin; while the stroma of iris contained blood vessels of different diameter forming a minor arterial ring of the iris—243.937 ± 41.1 µm and major arterial ring of the iris—395.648 ± 44.8 µm, and collagen fibers, spindle-shaped fibroblasts, macrophages containing phagocytized melanin, nerves and two well-developed smooth muscles: sphincter muscle (made of smooth muscle cells) and dilatator muscle (made of contractile extensions of myoepithelial cells of the inner layer of the posterior epithelium); the posterior surface of the iris is the retinal pigment layer (the iris part of the retina) made up of two layers of cells containing granules of melanin (Figure 4C–G). The pupil in these bears was big and round in shape. The visual part of the retina consisted of 10 layers arranged sequentially, starting from the pigment layer bordering the choroid: *stratum pigmentosum*, *stratum bacillorum et conorum*, *membrane limitans glie externa*, *stratum granulosum externum*, *stratum plexiforme externum*, *stratum granulosum internum*, *stratum pexiforme internum*, *startum fibrarum nervosarum et membrana limitans glie interna* (Figure 4H). The retinal pigment layer was a single-layer cubic epithelium (not seen in Figure 4H). The thickness of the visual part of the retina (excluding the pigment layer) in the Asiatic black bear was 122.32 ± 5.63 µm. The lens was a biconvex round body. The front pole of the lens facing the iris was more convex, while the rear pole of the lens facing the vitreous chamber of the eyeball was slightly convex. The anterior surface was covered with a single-layer cylindrical epithelium, which was not found on the posterior surface. The lens was surrounded by a thin capsule (15.045 ± 2.58 µm) and characterized by PAS strongly (+++) positive reaction, and the lens framework was elongated with lens fibers.

**Figure 5:**
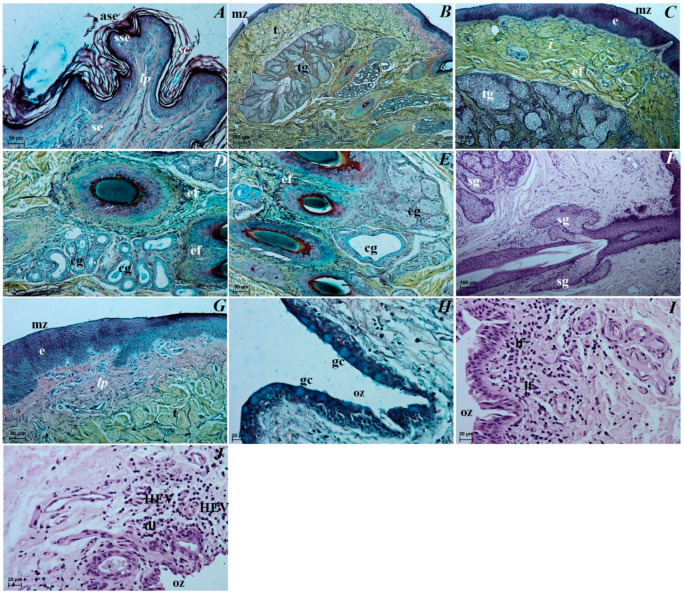
Photomicrograph of the *Ursus thibetanus* upper and lower eyelids. ase—anterior surface of eyelid, cg—ciliary gland, dl—diffuse lymphocytes, e—epithelium, ef—elastic fibres, gc—goblet cells, lf—lymphoid follicle, *lp*—*lamina propria*, HEV—high endothelial venules, mz—marginal zone, oz—orbital zone, *sc*—*stratum corneum*, se—stroma of eyelid, sg—sebaceous glands, sse—stratified squamous epithelium, t—tarsus, tg—tarsal gland. (**A**–**E**,**G**,**H**) = Movat pentachrome stain; (**F**,**I**,**J**) = H&E. Scale bars: (**B**) = 200 µm; (**A**,**C**,**E**,**G**) = 50 µm; (**D**,**H**–**J**) = 20 µm.

The upper and lower eyelids. The anterior surface of the upper eyelid and lower eyelid was covered by stratified squamous epithelium with five to eight layers of nucleated cells. The superficial layer of the stratified squamous epithelium was covered with a thick stratum corneum (57.369 ± 17.1 µm). The stratum basale epithelial cells lying on the basement membrane contained a small number of melanocytes. Under the epithelium lay a thick lamina propria formed by loose fibrous connective tissue, which was characterized by the presence of numerous elastic and reticular fibers (Figure 5A). Well-developed and heavily branched tarsal glands have been seen in both eyelids. In the upper and lower eyelids, a thick eyelid was seen that consisted of dense fibrous connective tissue (Figure 5B,C). The framework of the eyelids was dense connective tissue of irregular weaving containing very distinct elastic fibers, in which numerous and highly branched sebaceous glands and ciliary glands were arranged, the secretory sections of which could also be observed within the lamina propria (Figure 5C–F). The posterior surface of eyelids consisted of two parts: the marginal zone formed by the external border of the upper and lower eyelids and the orbital zone included part of the conjunctiva that contacted the eyeball. The marginal zone was covered by stratified columnar epithelium with nine to twenty layers of nucleated cells (Figure 5G). The epithelial cells of the stratum basale on this stratified columnar epithelium did not contain melanin granules. The orbital zone was covered by three to four non-keratinized layers of cells. In contrast, the orbital zone had seven to ten conjunctival folds containing a large number of goblet cells located within the non-lymphoid region (Figure 5H). The observed preparations in the orbital zone in the lymphoid region in both eyelids revealed the presence of lymphoid follicles, diffuse lymphocytes and HEV (high endothelial venules) located within the conjunctival folds (Figure 5I,J).

**Figure 6:**
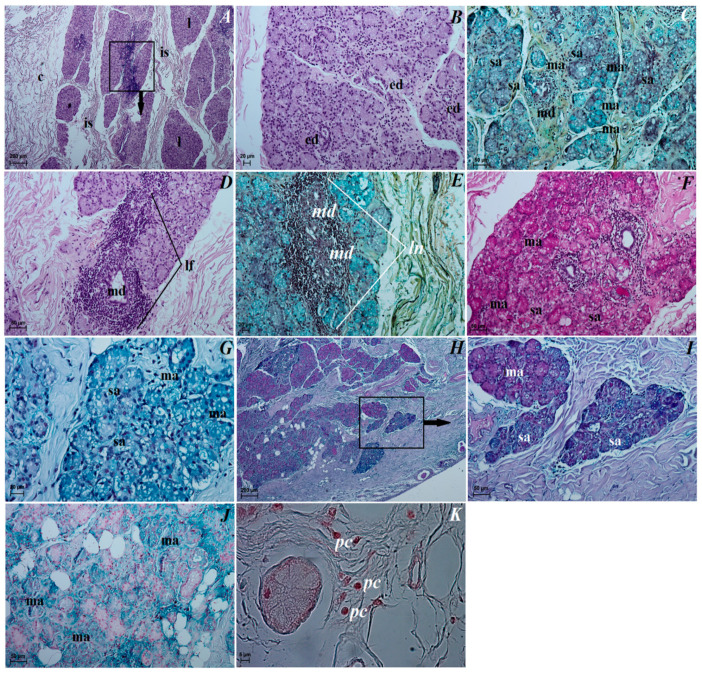
Photomicrograph of the *Ursus thibetanus* superficial gland of the third eyelid. (**A**,**B**,**D**) = H&E; (**C**,**E**) = Movat pentachrome stain; (**F**) = PAS strongly (+++) positive reaction in the mucous acini and slightly (−/+) positive reaction in the serous acini; (**G**) = AB pH 2.5 slightly (−/+) positive reaction in the mucous and serous acini; (**H**,**I**) = AB pH 2.5/PAS middle (++, magenta color—neutral mucins) in the mucous acini and weakly positive reaction (+, blue color—acidic sulfated mucosubstances and sialomucins) in the serous acini; (**J**) = HDI middle (++) positive reaction in the mucous acini and HDI negative (−) reaction in the serous acini; (**K**) = MGP Y stain. c—capsule, ed—excretory duct, is—interlobar septa, l—lobes, ln—lymphatic tissue, lf—lymphoid follicles, ma—mucous acini, md—main duct, pc—plasma cells, sa—serous acini. Scale bars: (**A**,**H**) = 200 µm; (**C**,**D**,**F**,**I**,**J**) = 50 µm; (**B**,**E**,**G**) = 20 µm; (**K**) = 5 µm.

The superficial gland of the third eyelid. These glands are characterized by a follicular multilobar branched complex structure with seromucous nature. This gland was surrounded by a big intraperiorbital fat body, under which was a thick connective tissue capsule. From this connective tissue capsule, both thick and thin interlobar septa, which divided gland structure into numerous big and small lobes, departed deeper into the gland (Figure 6A). The connective tissue capsule and interlobar septa were composed of dominant elastic fibers, interspersed with collagen and reticular fibers; numerous blood vessels and nerves were also observed. The excretory ducts located within the glandular stroma were composed of a cylindrical monolayer epithelium (Figure 6B). The lobes consisted of numerous serous acini containing a small lumen composed of conical cells with basophilic cytoplasm and with sparse mucous acini that contained a big lumen and were formed of tall conical cells with also basophilic cytoplasm (Figure 6C). Within the gland lobes, there were numerous main ducts built up from a monolayer cylindrical epithelium surrounded by lymphocyte clusters forming clustered lymph nodes (Figure 6D,E). The MGP Y staining revealed the presence of the few plasma cells located in the glandular interstitium (Figure 6K).

**Figure 7:**
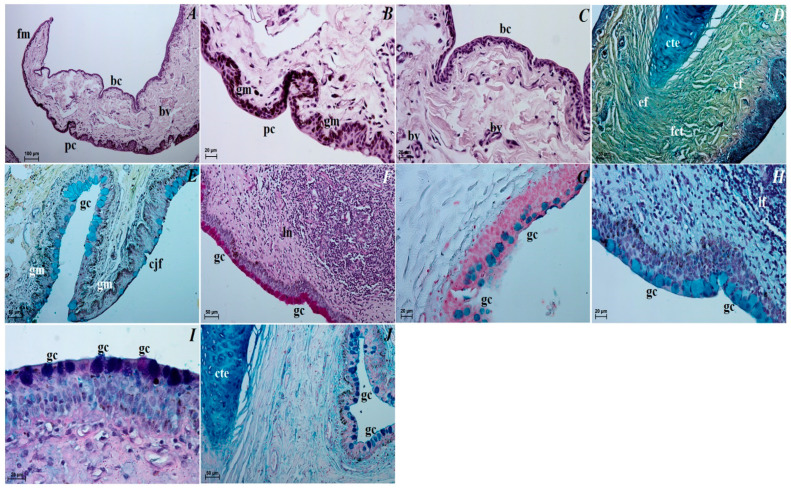
Photomicrograph of the *Ursus thibetanus* third eyelid. (**A**–**C**), = H&E; (**D**,**E**) = Movat pentachrome stain; (**F**) = PAS strongly (+++) positive reaction in the goblet cells; (**G**) = AB pH 1.0 strongly (++/+++) positive reaction in the goblet; (**H**) = AB pH 2.5 strongly (+++) positive reaction in the goblet cells; (**I**) = AB pH 2.5/PAS middle (++, magenta color—neutral mucins) positive reaction in the goblet cells; (**J**) = HDI strongly (+++) positive reaction in the goblet cells. bc—bulbar conjunctiva, bv—blood vessels, cf—collagen fibres, cjf—conjunctival fold, cte—cartilage of the third eyelid, ef—elastic fibres, fct—fibrous compact tissue, fm—free margin, gc—goblet cells, gm—granules of melanin, lf—lymphatic follicle, ln—lymphatic tissue, pc—palpebral conjunctiva. Scale bars: (**A**) = 100 µm; (**D**–**F**,**J**) = 50 µm; (**B**,**C**,**G**–**I**) = 20 µm.

The third eyelid. The palpebral conjunctiva of the third eyelid was covered by a multilayer squamous epithelium composed of five–eight layers of nucleated cells and numerous goblet cells, while the bulbar conjunctiva of the third eyelid contained a multilayered cubic epithelium consisting of three to four layers of epithelial cells with single goblet cells (Figure 7A–C). The free margin of the third eyelid had a large number of melanocytes and was composed of irregularly woven fibrous connective tissue containing numerous blood vessels of small diameter (Figure 7B–D). The cartilage of the third eyelid was surrounded by a thick layer of collagen and elastic fibers and was composed of hyaline tissue (Figure 7D). A large conjunctival fold was observed within the bulbar conjunctiva, which had a subepithelial tissue conjunctival lymphoid follicle underneath (Figure 7E,F).

**Figure 8:**
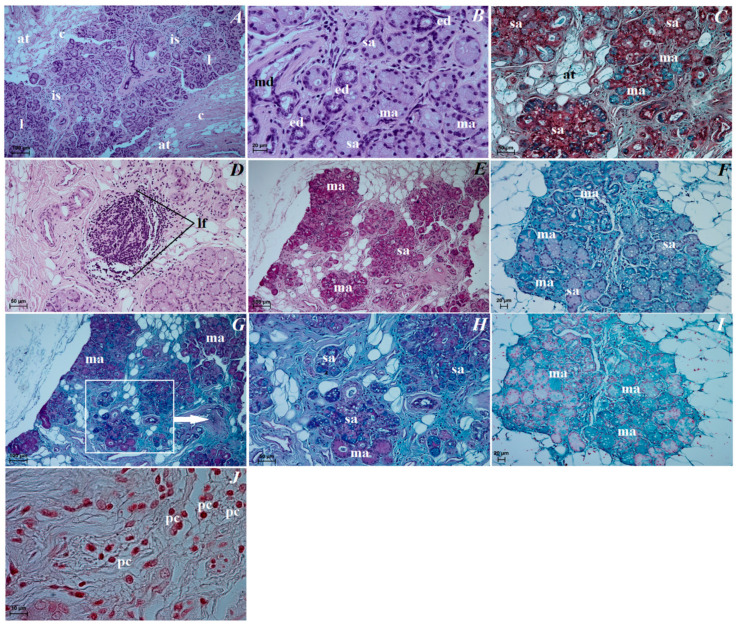
Photomicrograph of the *Ursus thibetanus* lacrimal gland. (**A**,**B**,**D**) = H&E; (**C**) = Movat pentachrome stain; (**E**) = PAS middle (++) positive reaction in the mucous acini and weakly (+) positive reaction in the serous acini; (**F**) = AB pH 2.5 middle (++) positive reaction in the mucous and negative (−) reaction in the serous acini; (**G**,**H**) = AB pH 2.5/PAS middle (++, magenta color—neutral mucins) in the mucous acini and middle (++, blue color—acidic sulfated mucosubstances and sialomucins) in the serous acini; (**I**) = HDI weakly (+) positive reaction in the mucous acini and HDI negative (−) reaction in the serous acini; (**J**) = MGP Y stain. at—adipose tissue, c—capsule, ed—excretory duct, is—interlobar septa, l—lobes, lf—lymphoid follicles, ma—mucous acini, md—main duct, pc—plasma cells, sa—serous acini. Scale bars: (**A**,**E**,**G**) = 100 µm; (**C**,**D**) = 50 µm; (**B**,**F**,**H**,**I**) = 20 µm; (**J**) = 10 µm.

The lacrimal gland. These glands were a multilobar acinar branched complex structure producing a mucosal–serous secretion. This gland was surrounded by a thick connective tissue capsule, interspersed with numerous fat cells, collagen and reticular fibers, which, penetrating deep into the gland, formed poorly marked flaps through narrow interlobar septa (Figure 8A). The excretory ducts were lined, as well as in the superficial gland of the third eyelid, with a cylindrical monolayer epithelium located between the glandular units (Figure 8B). The lobes were formed from two types of secretory cells: dominating mucous acini formed from tall conical cells with a small lumen or big lumen, and with vacuolized and pale cytoplasm and with sparse serous acinus (conical cells with a small lumen and eosinophilic cytoplasm) (Figure 8B,C). A single small lymphoid follicle was observed within the glandular interstitium and the presence of numerous plasma cells in the glandular interstitium (MGP Y stain) was revealed (Figure 8D–J).

### 3.4. Histochemical Observation of the Eyelids and Orbital Glands

**Figure 9:**
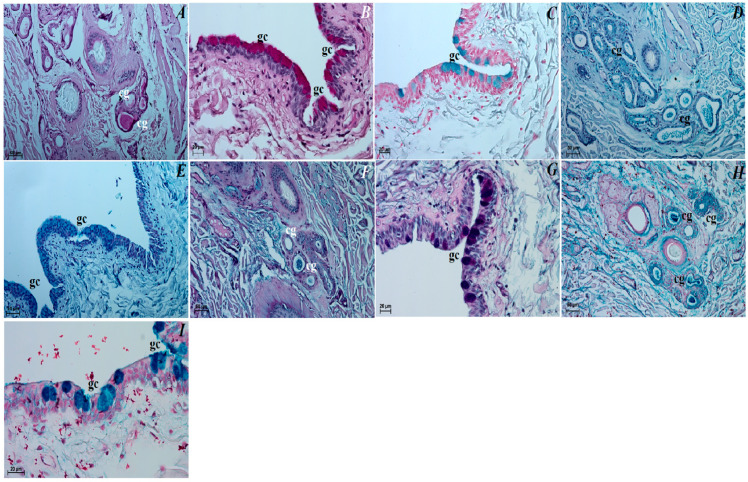
Histochemical photomicrograph of the *Ursus thibetanus* upper and lower eyelids. (**A**) = PAS slightly (−/+) positive reaction in the ciliary glands (cg); (**B**) = PAS strongly (+++) positive reaction in the goblet cells (gc); (**C**) = AB pH 1.0 strongly (+++) positive reaction in the goblet cells; (**D**) = AB pH 2.5 slightly (−/+) positive reaction in ciliary glands; (**E**) = AB pH 2.5 strongly (+++) positive reaction in the goblet cells; (**F**) = AB pH 2.5/PAS slightly (−/+, blue color—acidic sulfated mucosubstances and sialomucins) positive reaction in the ciliary glands; (**G**) = AB pH 2.5/PAS strongly (+++, magenta color—neutral mucins) positive reaction in the goblet cells; (**H**) = HDI slightly (−/+) positive reaction in the ciliary glands; (**I**) = HDI strongly (+++) positive reaction in the goblet cells. Scale bars: (**A**,**D**–**F**,**H**) = 50 µm; (**B**,**C**,**G**,**I**) = 100 µm.

The upper and lower eyelids. In both eyelids, there was a negative PAS, AB pH 1.0, AB pH 2.5, AB pH 2.5/PAS and HDI reactions in the tarsal and sebaceous glands (see Table 1). The ciliary glands characterized weakly (−/+) PAS, AB pH 2.5 and AB pH 2.5/PAS (blue color) positive reaction (Figure 9A,F, see Table 1). The AB pH 1.0 staining of the ciliary glands was rated as (−) (negative reaction), and HDI middle (++) positive reaction (Figure 9D,H, see Table 1). The PAS, AB pH 1.0, AB pH 2.5, AB pH 2.5/PAS (magenta color) and HDI stains revealed a strongly positive reaction (+++) in the goblet cells in the palpebral and bulbar conjunctival epithelium (Figure 9B,C,E,G,I, see Table 1).

The superficial gland of the third eyelid. The serous acinar cells showed a PAS and AB pH 2.5 weakly (−/+) reaction, AB pH 1.0 and HDI negative (−) reaction, while AB pH 2.5/PAS (blue color) weakly (+) positive reaction. For mucous acinar cells of these gland, PAS, HDI and AB pH 2.5/PAS (magenta color) middle positive (++) reaction, and AB pH 2.5 slightly (−/+) positive reaction were found (Figure 6F–J, see Table 1). The AB pH 1.0 stain in these mucous acini was a negative reaction (see Table 1).

The third eyelid. The goblet cells of the third eyelid showed a strongly (++/+++ or +++) PAS, AB pH 1.0, AB pH 2.5, AB pH2.5/PAS (magenta color) and HDI positive reaction (Figure 7F–J, see Table 1). 

The lacrimal gland. Staining of the serous acinar cells using the PAS method demonstrated the presence of a weakly positive reaction (+). The AB pH 1.0, AB pH 2.5 and HDI staining techniques proved the presence of negative (−) reaction in these cells. The AB pH 2.5/PAS method revealed a blue reaction in the serous acini rated as ++. The numerous mucous acinar cells in these glands gave a positive reaction, which was rated as ++ using the PAS, AB pH 2.5, AB pH 2.5/PAS (magenta color) and HDI stains. These mucous cells characterized negative reaction using AB pH 1.0 stain (Figure 8E–I, see Table 1). 

## 4. Discussion

The Ursidae family arose early in carnivoran evolution from Miadicae—a small tree-climbing common ancestor (approximately 33–37 million years old)—and together with the Canidae, is thought to be one of the most ancient families within the Caniformia [3,11]. Currently living eight species of bear are divided into the following three subfamilies: Ailuropodinae (giant panda), Tremarctinae (Andean bear) and Ursinae (“typical bears”—American black bear, brown bear, polar bear, Asiatic black bear, sloth bear and sun bear). The Andean bear and giant panda are the most ancient and distinctive species [11]. For the other six species, the inter-relationships are poorly understood, and as Hunter, 2011 [11], reports, it is known that polar bears recently evolved from a brown bear population isolated during the mid-Pleistocene transition (about 200,000 years ago). Unfortunately, there are fewer studies concerning the exact orbit and eye anatomy, or studies in the field of veterinary ophthalmology, in Ursidae, Musteloidea or wild Canidae compared to a domestic dog or Pinnipedia. We hope that the presented research results significantly expand the existing knowledge on comparative anatomy in the orbit, eye tunics and chosen accessory organs of the eye in the family Ursidae.

### 4.1. The Eyeball and Eye Tunics

The obtained results of the eyeball macroscopic measurements performed in the examined Asiatic black bear were similar to the brown bear, American black bear *Ursus americanus*, grizzly bear *Ursus arctos horribilis* and sloth bear *Melursus ursinus* [26,66], but also they were similar to the measurements made on some representatives of the Canidae family (crab-eating fox *Cerdocyon thous*, Arctic fox *Alopex lagopus* and bush dog *Speothos venaticus*) compared to mongrel dogs, South African painted dog *Lycaon pictus*, wolves *Canis lupus*, gray fox *Urocyon cinereoargenteus* and red fox *Vulpes*, where the eyeball measurements were characterized by much higher parameters [25,66,67,68]. However, some species of Mustelidae and the only representative of Ailuridae red panda *Ailurus fulgens* [25,66,69] are characterized by small and medium dimensions of the eyeball compared to Ursidae and Canidae. The greatest differences in the dimensions of the eyeball within the Canidae family will be characteristic of domestic dogs, and in particular purebred dogs, and most likely it will be related to differences in craniometry, the type of skull (dolichocephalic, mesocephalic and brachycephalic) and the dimensions of the eye socket, which in the case of a domestic dog are correlated with the size of the animal [68,70]. According to Heard-Booth and Kirk, 2012 [66], the size of vertebrate eyes depends on body or head size, diet and activity pattern. Interestingly, it has also been suggested that the speed of movement affects the size of the eyeball and is a relationship, as per Leuckart’s law [66]. Leuckart’s law suggests that animals capable of attaining high locomotor speeds must have large eyeballs to improve visual acuity and avoid collisions with obstacles in the way of the animal’s movement [71,72,73,74]. The research conducted by Heard-Booth and Kirk, 2012 [66], on 50 species from 10 mammalian orders showed, by Leuckart’s law, that the absolute size of the eyeball is significantly positively correlated with the maximum speed of movement of the tested mammals. These results also show that faster-moving mammals have larger eyeballs than their slower-moving close relatives. These authors come to an interesting conclusion that the maximum speed of movement of animals is one of several very important selection factors that may have influenced the evolution of the size of the eyeball in animals. According to Brown, 2009 [3], the bears were characterized by the unusually small eyeballs compared to the overall body size; however, in analyzing the results of the Heard-Booth and Kirk, 2012 [66], study, also conducted on *Ursus americanus* and *Ursus arctos* craniometry, bears’ diet, activity pattern, and speed increase in the range of 47–48 km/h, we see that these animals have small eyeballs. According to Davis, 2019 [75], in the case of Phocidae and Otariidae (Pinnipedia), because they are not capable of active echolocation, the size of their eyeballs is similar to nocturnal terrestrial mammals. These families have a large eyeball, where the interorbital width is reduced, and the jugal bone that forms the anterior zygoma is curved ventrally to enlarge the cranial orbit, furthermore, in the Pinnipedia, the visual field of each eye overlaps enabling binocular vision and stereopsis [75]. It is interesting that the walrus *Odobenus rosmarus*, like Phocidae and Otariidae, is not capable of echolocation, but his eyeballs are ellipsoidal in shape and are extremally small both in absolute size and in relative size to the body compared to other pinnipeds, and are located dorsolaterally on the head, which limits the binocular vision [75,76]. In the case of amphibious marine carnivores (Pinnipedia and sea otter) where their eyeball is believed to be adapted for underwater and aerial vision, they also have remarkable morphological and functional specialization for both habitats. As reported by Piggins (1970), cited by Sivak et al., 1989 [77], and Hanke et al., 2006 [78], in the harbor seal *Phoca vitulina*, harp seal *Pagophilus groenlandicus* and the Weddell seal *Leptonychotes weddelli*, the eyes feature a high degree of astigmatism in the air, which results from corneal curvature and much of it disappears when the eye is underwater [79]. Jamieson and Fisher, 1971 [80], and Jamieson and Fisher [81] suggest that astigmatism is a side-effect of ocular streamlining underwater or, as reported by Sivak, 1980 [78], and Walls, 1963 [82], was incorporated in a model for amphibious vision defined as “inactive accommodation”. Mass and Supin, 2007 [83], reported that the semiaquatic mammals are characterized by emmetropia or refraction of light to focus on the retina, while submerged in water, and most have mechanisms to achieve emmetropia above water to prevent myopia over the water surface.

The maximum and minimum corneal diameter in the examined Asiatic black bear was similar to the other Ursidae species (*Ursus americanus*, *Ursus arctos*, *Melursus ursinus*) as well as in some wild Canidae species (*Alopex lagopus*, *Cerdocyon thous*, *Urocyon cinereoargenteus*) or in Procyonidae (*Bassariscus astutus*, *Procyon lotor*) [25]. Histological studies revealed that the cornea in the examined Asiatic black bear consists of only four layers because Bowman’s membrane was not found, as well as in domestic dogs, wolves, dingo *Canis dingo* and corsac fox *Vulpes corsac* [84,85], while it occurs in the South African painted dog [68]. In the Asiatic black bear, the anterior corneal epithelium consists of 5–6 layers of nucleated cells in the peripheral part and 6–7 layers of cells in the axial part, where a similar number of layers could be observed in *Canis dingo*, *Canis familiaris*, *Cerdocyon thous* and *Vulpes corsac* [86], while *Canis lupus* and *Lycaon pictus* were slightly higher [68,85,86,87,88]. Nautscher et al., 2016 [85], showed in their studies that the number of cell rows correlates with the corneal thickness, and that the thickness of the cornea changes with the age of the subject [85]. The results of histometric measurements of the corneal layers in Asiatic black bear showed similarity to some wild Canidae (*Canis dingo* and *Vulpes corsac*), while higher values of these dimensions occurred in the domestic dogs and South African painted dogs [68,84]. In the pinnipeds, the cornea is characterized by a central flattened stripe in the vertical meridian and the near-spherical shape of the eyeball [84,89,90], as well as in the sea otter *Enhydra lutra*, where the eyeball is similar to those terrestrial mammals—it is almost spherical and the axial eye length is only a little shorter than the diameter, but the corneal curvature has very low convexity [83]. As reported by Mass and Supin, 2018 [89], of all tested pinnipeds species, only in the Steller sea lion *Eumetopias jubatus* is the cornea in the central part characterized by low convexity and with no truly flat corneal emmetropic window. Because the corneal curvature in the seals, sea lions and walrus is poorly marked and the central part of it is absent, the outer surface of the cornea is minimal and does not function as a refractive unit in air. Due to the small curvature of the cornea and the similar refractive index of the cornea and water, the cornea does not function as a refractive unit in water. Therefore, changes in the media in front of the cornea, from air to water or from water to air, have a minimal or no effect on the position of the focused image [89]. It is interesting that, according to Gaiddon et al., 1991 [90], large breed dogs are characterized by a slightly flatter cornea (a larger radius of curvature) than that of small or medium breed dogs.

Histologically the cornea in the Antarctic Weddall seal *Leptonychotes weddellii* consists of 5 layers and the corneal thickness described in pinnipeds was similarly (harp seal *Pagophilus groenlandicus*, California sea lion *Zalophus californianus*, Caspian seal *Pusa caspica*, Baikal seal *Pusa sibirica*, Straitopeller seal *Pusa sibirica*, Stallopias sea seal *Pusa sibirica*, Southern fur seals Arctocephalus sp and walrus) [89,91,92], and in the sea otter, the cornea had only four layers because, as is examined, Asiatic black bear Bowman’s membrane is absent, while in the sea otter the anterior corneal epithelium was extensively developed [70,91].

Our research showed, similarly to South African painted dogs, domestic dogs, cats, rats and humans within corneal limbus epithelium, the presence of palisades of Vogt, composed of conjunctival folds, containing niches for limbal epithelial stem cell (LESC) [93]. The LESCs are responsible for the regeneration of the corneal surface and help maintain its transparency. These cells are found only in Vogt’s palisades, which create a special microenvironment for their renewal and proliferation. According to Dua, 1998 [94], and Dua et al., 2000 [95], in the human deficiency of these cells leads to corneal opacification through conjunctivalization and vascularization of the transparent cornea. The LESC deficit in humans is influenced, in addition to genetic diseases, by chemical, heat or radiation burns and chronic inflammatory, while in the case of domestic and especially wild mammals, the incidence of conjunctivalization in corneal disease is difficult to define, because the methods that demonstrate the presence of conjunctivalization, such as impression cytology, are not commonly employed in veterinary ophthalmology [93].

In the examined terrestrial caniforms, the pupil was characterized by different shapes and for example, the pupil in our examined Asiatic black bear was big and round in shape, similar to that of other bear species subfamily Tremarctinae and Ursinae and also in the domestic dog and in some wild Canidae (*Chrysocyon brachyurus*, *Canis lupus*, *Lycaon pictus pictus*) [68,96], and according to Kirbas Dogan et al., 2020 [26], the brown bear *Ursus arctos horribilis* the pupil had a wider temporonasal length, while vertically slit-shaped in the giant panda, *Cerdocyon thous* and *Vulpes* [3,67,86,96,97] and in North American racoon *Procyon lotor* as nocturnal animals the pupil is wide [98]. Johnson, 1901 [24] reported that the pupil in Ursidae is round except *Ursus americanus*, in which it is vertically oval, dilatating to a full circle. McKay Strobel et al., 2020 [99], reported that the sea otter pupil size is smaller relative to other amphibious marine carnivores when accounting for test conditions. These authors suggest that sea otters have retained features for low-light vision, but rapid adjustments and acute underwater vision may be constrained across varying light levels by a combination of pupil shape, absolute eyeball size, and the presumed coupling between anterior lens curvature and pupil size during accommodation. The presence of in the giant panda vertical slit pupil compared to other bear species, as reported by Brown, 2009 [3], may possibly be related to improved night vision, but Hu, 2001 [100], reports that the giant panda’s eye contains stamens and cones, the stamens outnumbering cones, suggesting that giant pandas have sharp night vision while being capable of daylight and color vision. Giant pandas are not typically nocturnal mammals; they appear to be polycyclic animals, with the greatest activity occurring in the early morning and late afternoon [101]. Due to such variable pupil shapes, in the terrestrial mammals, as reported by Banks et al. [102] and Malmström and Kröger, 2006 [96], the shape and size of the pupils in these mammals depends on the habitat of a given species or family membership and activity periods, hence its adaptation to multifocal optical systems and whether they are “predators” or “prey” (for herbivore (prey) animals, daytime predators and, as in the nocturnal predators, diurnal–nocturnal predators and crepuscular vertebrates to reach maximum light-gathering ability) [68]. As reported by Mass and Supin, 2007 [83], most pinnipeds have pear-shaped pupils when constricted, the exceptions being the bearded seal *Erignathus barbatus*, which has a diagonal pupil, and the California sea lion, which has a pupil shaped like a tear-drop [91]. In the walrus, the pupil is oval during low light conditions, and when the light level increases, the pupil is ventrally narrower, taking the shape of a keyhole [76]. The Phocidae and Otariidae have a round pupil when dilated, and when it narrows it is a vertical slit [24,75]. In the pinnipeds and the sea otter-like mammals, similar to that in the examined Asiatic black bear, the ciliary muscle, the dilatator muscle and the sphincter muscle is well-developed [70,75,76,83,103,104], although in Pinnipedia, accommodation is either absent or very weak [75,83]. However, according to Welsch et al., 2001 [92], in the Antarctic Weddell seal, the ciliary muscle was poorly developed (the structure of these muscles was surprisingly loose and comprises rather few muscle cells), thus the ability of accommodation is relatively poor.

The tapetum lucidum is a specialized reflective layer of the choroid, which is designed to increase the ability of the retina to function under low light levels and increase photoreceptor layer stimulation by intrinsic fluorescence of its structure when stimulated by incident light [105]. Macroscopically, the tapetum lucidum in the examined Asiatic black bear was similar in shape to dark brown opaque semicircle (crescent), while according to Kirbas Dogan et al., 2020 [26], in the brown bear this tapetum was yellow-green color and triangular shape. The different degree of coloration of tapetum lucidum is due to the optical phenomenon of thin-film interference rather than the presence of specific pigments (that is, tapetal coloration is structural rather than pigmentary). Murphy et al., 2012 [105], reported that in the domestic dogs, the tapetal coloration, as well as the degree of pigmentation present in the non-tapetal region of the fundus, is correlated with coat color (e.g., dogs with brown and red coat colors had a more orange-tinted tapetal color while dogs with white or gray coats more often had a green-colored tapetal region) but also the tapetal size was in some cases correlated with breed with the smaller breeds often having proportionally smaller areas of the fundus occupied by tapetum. In the case of wild Canidae, a large color variation was also observed of this tapetum lucidum, not only between species, but also within the same species (green or yellow with a green border in the crab-eating fox, yellow with a green border in the maned wolf, or milky in the South African painted dog) [67,68,97] and, for example, on the kinkajou *Cercoleptes caudivolvulus* (Procyonidae), the tapetum lucidum is a yellowish-green color, while on the ringtailed coati *Nasua rufa* (Procyonidae) it is bright green with yellowish patches [24]. The variation in the color of the tapetum lucidum can also be observed in semiaquatic mammals; for example, in the sea otter, it has a semicircular shape and the central tapetal area is blue-green and lightly mottled with yellow, while the intermedia area is yellow heavily mottled with orange [99], while in the California sea lion it was green [91]. Histologically, the tapetum lucidum in the examined Asiatic black bear is similar to the tapetum lucidum cellulosum in domestic and wild Canidae, Pinnipeds and Mustelidae [68,76,91,92,99,106,107,108,109,110,111,112]. The characteristic feature of this tapetum is the variable number of cell layers depending on the species of the animal, but also, as reported by the above-mentioned authors, a different number of cell layers was observed between the domestic dogs. Mass and Supin, 2007 [83], cited by Walls, 1942 [71], reported that in the pinnipeds (except the walrus, where tapetum lucidum is absent [75]) the tapetum lucidum cellulosum is one of the best developed among the terrestrial and aquatic mammals and is formed with intracellular reflective rodlets [106]. In the Canidae, the number of cell layers ranged from 9 to 20 [106,107,108,109,110,111,112,113], while in the Pinnipedia it oscillated between 12 and 35 [76,111], in the sea otter this tapetum comprises 7 to 8 layers of cells [99], and in the ferret *Mustela putorius* it comprises 5 to 7 cell layers [111]. In our Asiatic black bear, we observed that compared to the family Canidae and Pinnipedia, this tapetum lucidum was composed of a very small number of layers of cells (2–3 layers of cells) that showed degenerative features. The same number of cell layers was observed in the ranch mink Mustela vision, but also in this species degeneration features of wallpaper cells (non-regular internal arrangement of membranes) [106,111]. Ollivier et al., 2004 [111], report that the cause of this wallpaper degeneration is unknown but could be related to dietary insufficiencies in specific animals, or perhaps the genetics of inbreeding. In the case of our test bear, we suppose that the cause of the degenerative changes in the wallpaper cells may be related to the age of the individual, but these changes may also have resulted from an inflammatory process within the maxillary sagittal arch, but perhaps it is a feature of the Ursidae family; therefore, precise determination of it requires further research in the Ursidae. Another factor could be improper light exposure in captivity, what can cause eye damage in zoo animals.

The lens of the eye is soft, transparent and was protein-rich [105]. In the examined Asiatic black bear, the lens was biconvex round like in the sloth bear and in domestic and wild Canidae structure, where it is suspended in contact with the posterior face of the iris and the anterior face of the vitreous body [33,67,68,105]. Its function is to bring images into critical focus on the photoreceptor layer of the retina. In the sea otter, the lens is lenticular; however, the front surface of the lens has a protuberance of increased curvature in opposite to pinnipeds and cetaceans where it has a spherical shape [75,78,83,91,92]. According to Murphy et al., 1990 [70], cited by Mass and Supin, 2007 [83], a characteristic feature of the eyeball in the sea otter is the attachment of the iris to the anterior surface of the lens, which in turn is associated with contraction of the iris muscles and thus a change in the curvature of the anterior lens surface. Such a mechanism can provide a range of accommodation up to 60 diopters, thus compensating for the appearance of refraction on the surface of the cornea in the air and its disappearance in water, and consequently allows emmetropy in both air and water [70,75,96]. Malmström and Kröger, 2006 [96], suggest that in the terrestrial mammals the lens type and its relation to the pupil is an important factor for adaptation to multifocal optical systems, for example, for nocturnal and crepuscular vertebrates to reach maximum light-gathering ability.

In our Asiatic black bear, the retinal photoreceptor layers consisted of rods and cones, as was seen in the brown bear *Ursus arctos horribilis*, diurnal and nocturnal Canidae, and Mustelidae [23,26,68,70,83,114]. Research by Peichl et al., 2005 [27], and Heyward et al., 2020 [23], on the American black bear, brown bear and the polar bear showed the presence in retinal photoreceptor L-cones and S-cones in the brown bear and polar bear, and long/medium (L/M) wavelength sensitive L/M-cones and S-cones in the American black bear, suggesting that the bears have the potential for dichromatic color vision with high acuity, similar to the red fox and Arctic fox, which possess a majority of middle-to-longwave-sensitive (M/L) and a minority of shortwave-sensitive (S) cones, indicating dichromatic color vision [115]. The polar bear, which is overall well adapted to its semiaquatic lifestyle and its rather colorless habitat of snow and ice, has also retained both cone opsins and points to different visual demands and adaptive pressures on polar bears and seals. Overall, cone densities and S-cone proportions in the Ursidae studied are higher than in most other mammals, indicating an adaptation to diurnal vision [27]. According to Mass and Supin, 2007 [83], the retinal structure of the pinnipeds is similar to terrestrial mammals, but there are several features unique to aquatic mammals. In the nocturnal terrestrial carnivores, like in all pinnipeds, the layer of visual (receptor) cells consists predominantly of rods, which were characterized by slender, nearly cylindrical, long outer segments densely packed, and the outer limiting membrane is discernible between the photoreceptor and outer nuclear layers. The outer nuclear layer is composed of receptor perikarya arranged in a multilevel manner, and the inner nuclear layer is thin and rather chaotically organized, while the ganglion layer consists of a single row of rather large ganglion cells separated by wide intercellular spaces [83,116,117,118,119,120]. A 1970 study by Landau and Dawson [121] and Nagy and Ronald [122] on the pinnipeds found no cones of the retina, but more detailed studies by Jamieson and Fisher (1971) [83] and Nagy and Ronald (1975) [122] on the harbor seal and harp seal showed the presence of rods and cones, wherein cones accounted for approximately 1% of the photoreceptors [83]. Research by Peichl et al. (2001) [123] showed that the several pinniped species only have L/M subtype cones and therefore monochromatic vision. However, in the case of the sea otter, the retina is characterized by more properties in common with terrestrial mammals than the aquatic mammals, i.e., the majority of ganglion cells in the sea otter’s retina are rather small, contrarily to aquatic and similarly to terrestrial mammals, meaning that cell size in the high-density streak in the sea otter’s retina is also close to that of terrestrial mammals; a proportion and distribution of these three groups in the sea otter are close to those of α-, β-, and γ-cells in the ferret [83,124,125]. Research by Jacobs and Deegan II, 1992 [126] on family Procyonidae using electroretinogram (ERG) flicker photometry defining cones photopigments showed that the raccoon *Procyon lotor* and kinkajou are monochromatic (nocturnal species), while the coati Nasua nose is dichromatic (diurnal species).

### 4.2. The Orbital Region

Unfortunately, due to the presence of only one individual, we were not able to measure the orbit with craniometry and compare the obtained test results with the studies on other bear species, and therefore we focused only on the anatomical description of the orbital area. The detailed anatomical description of the orbital region in the examined Asiatic black bear, and Sloth bear [33], was similar to the domestic and wild Canidae, but several characteristics of only family Ursidae were observed. Common features for both families were an open orbit composed of the same bony structures [33,57,67,68], with no supraorbital foramen, trochlear fovea for the superior oblique muscle and fossa of ventral oblique muscle being found, while there was presence of optic canal, orbital fissure, foramen rotundum, rostral and caudal alar foramen with the alar canal [32,57,67,68,127]. The supraorbital foramen was also absent in the badger *Meles meles*, marten *Martes foin* and otter *Lutra lutra* [128,129], while very well-developed infraorbital foramen is present in the otter and badger, less checked in the raccoon dog *Nyctereutes procyonides*, and not present in our examined Asiatic black bear [129,130]. The open-type eye socket was also found in the family Odobenidae, Otariidae and Phocidae [131,132,133]. The differences we observed between Canidae and Ursidae were single ethmoid foramen present in Asiatic black bear and South African painted dog, while it was double in domestic dogs; then, there was presence of a lacrimal sac in our Asiatic black bear and domestic dogs, but this was not present in the South African painted dog [33,49,67,68]. A characteristic feature of only the Ursidae family is a very clearly marked orbitotemporal crest, presence of rostral and caudal lacrimal processes. Research by Casares-Hidalgo et al., 2019 [134], showed that there is not a clear association between orbit orientation and the ecology of living carnivorans. These authors hypothesize that the evolution of the orbit in mammalian carnivores represents a new case of an ecological bottleneck specific to carnivorans.

Scant or virtually no scientific reports on the morphology of the accessory eye organs in the Ursidae family, and also in parvorder Musteloidea, forced us to compare our results with the Canidae and parvorder Pinnipedia families.

### 4.3. The Upper and Lower Eyelids

Macroscopic studies in the upper and lower eyelids in Asiatic black bear showed absent eyelashes in the anterior palpebral margin in the lower eyelid, similarly to the domestic dogs and wild dogs (South African painted dog and crab-eating fox) [67,68,135], while Carvalho et al., 2020 [97], reports that in the maned wolf and crab-eating fox the lower eyelid the eyelashes were short and scarce accessory eyelashes. The situation is different for seals, sea lions and walruses, where eyelashes have vanished and eyelids have become modified as well, and to compensate for this lack of eye protection, the pinnipeds constantly produce copious amounts of eye mucus [136]. Histologically, both eyelids in our Asiatic black bear were structurally similar—characteristic features were the highly developed and numerous tarsal glands (also known Meibomian glands), sebaceous glands and ciliary glands, the secretion of which enters the superficial oily layer of the precorneal tear film, similarly for Canidae [67,68,97] and for the domestic ferret *Mustela putorius furo* [137]. According to Gulland et al., 2018 [138], the pinnipeds do not have a tarsal gland in their eyelids, but do have a small associated sebaceous gland in the outer eyelids skin at the eyelid margin; nevertheless, discharge of these glands do not contribute to the precorneal tear film [139]. Collitz et al., 2012 [140], described that these glands in the pinnipeds are different in size and orientation compared to terrestrial mammal tarsal glands, and due to the lack of lipid components, the main role in the protection of the eyeball is played by aqueous and mucous components included in the precorneal tear film. Dartt, 2011 [141], points out that in pinnipeds, the mucous layers are thicker than terrestrial mammals, but this layer is still the thickest in the Cetacea, due to only water habits. Another characteristic of the Asiatic black bear is the presence of numerous conjunctival folds on the ocular zone with numerous goblet cells. However, according to Welsch et al., 2001 [92], in the Antarctic Weddell seal, the bulbar conjunctiva were present as single goblet cells and forms irregular folds and crypts. As reported by Davidson and Kuonen, 2004 [142], Moore et al., 1987 [143], Moore and Tiffany, 1979 [144], and Knop and Knop, 2005 [145], distribution density and the number of the goblet cells at bulbar conjunctiva was different between species (human, different domestic animals, rodents, monkey, birds) and affects higher mucin production and consequently ocular surface protection (especially the corneal immune protection) by trapping particulate debris and bacteria and contributes to local immunity by adherence IgA and lysozyme [146]. Our research also showed that the presence in both eyelids of structures of the CALT in the form of lymphoid follicles, diffuse lymphocytes and specialized high endothelial venules permit the lymphocyte migration and exchange between eyeball tissue and other organs of the MALT, as well as in Canidae [67,68,147]. In the domestic dog, as reported by Wenzel-Hora et al., 1982 [148], the number, size and localization of the lymphoid cells is related to the age of the animals but also influenced by antigenic stimulation. We suppose that in our Asiatic black bear, the presence of so many CALT components probably could have had an impact on the age of the individual, but also with the ongoing inflammatory process in the area of the maxillary alveolar arch. Any changes in corneal, eyelid or eyeball dysfunction caused by ocular surface diseases (OSDs) including dry eye disease, meibomian gland distinction, allergic keratoconjunctivitis, rosacea, blepharitis, drug-induced toxicity, chemical and thermal burns, chronic use of contact lenses, and immunological diseases have an impact on the correct vision process, but also on the quality of life of animals [149].

### 4.4. The Superficial Gland of the Third Eyelid with the Third Eyelid

Our studies have shown that this gland on the Asiatic black bear, just like in wild and domestic Canidae, is located in the medial corner of the eye, between medial straight and ventral straight muscles, and was partially covered by the ventral oblique muscles [67,68,105,135,150,151]. Similarly to the South African the painted dog, the gland was oval in shape and light pink in color [68], in contrast to the domestic dog, where it had a tear-drop shape and was pink [105]. Morphometric studies showed that the superficial gland of the third eyelid was small, but interestingly, comparing the width measurement in the Asiatic black bear and South African painted dog to domestic dog and crab-eating fox, it was much wider in the two earlier species [67,68]. Because there is also only one male in our research, we cannot refer to whether sexual dimorphism may affect the size of the gland in Ursidae as it is in the case of mongrel dogs [152]. Histological analysis of the Asiatic black bear superficial gland of the third eyelid showed that it is a branched complex multilobar gland that produces a seromucous character secretion, compared to Canidae, where the gland had multilobar tubuloacinar structure in the domestic dog and also produces serous mucus secretions, while in the South African painted dog serous secretion was produced comprising an aqueous layer of the precorneal tear film [68,105,150]. At the same time, the question arises whether such a structure of the lacrimal gland in the Asiatic black bear is a species feature, or whether it is a subfamily or perhaps a characteristic feature for the entire Ursidae family. In the case of parvorder Pinnipedia on the sea lion, this gland is referred to as the medial ocular gland or nictitating gland, which was located medially and was associated with the inner aspect of the third eyelid, where, similarly to temporal ocular gland, it had tubulo-acinar arrangements and potentially produced an aqueous component of the tear [139]. Interesting is the fact, as reported by Kelleher Davis et al., 2013 [139], that there are significant differences in the structure and localization of the eyeball glands between pinnipeds and Cetacea, which, as the authors suggest, is associated with a different degree of adaptation of marine mammal species to the aquatic environment (pinnipeds lead a partially aquatic lifestyle, and Cetacea are their fully adapted to aquatic lifestyle). Studies by Tarpley and Ridgway, 1991 [153], and Funasaka et al., 2010 [154], on the Cetacea (Atlantic bottlenose dolphin and baleen whales) showed that the orbital gland, also referred to by Rodrigues et al., 2015 [155] as the conjunctival gland, was an irregular multilobulated mass which surrounds the eyeball in a belt-like fashion on the corneal side of the globe’s transverse equator, compared earlier to the sea lion producing copious amounts of tears [139]. Kelleher Davis et al., 2013 [139], suggest that differences in tear volume may reflect the varying degrees of adaptation of marine mammalian species to an aquatic environment. Kelleher Davies et al., 2013 [139], while conducting on the sea lions and seals with the use of mobile interferometry, asked themselves whether pinnipeds have a lipid layer on the outer surface of the precorneal tear film. It transpired that the superficial oily layer of the precorneal tear film typically observed in terrestrial mammals is undetectable in pinnipeds, at least over the time course of this examination. Studies by Colitz et al. [140,156] and Kelleher Davis et al., 2013 [139], indicate that, unlike terrestrial mammals, precorneal tear film in the pinnipeds consists of mucous and aqueous components, although there is a sebaceous gland in the eyelids. However, these do not contribute to the precorneal tear film.

The third eyelid in the test Asiatic black bear was T-shaped, similarly and previously described in the domestic and wild Canidae, and it was very pigmented and located in the medial canthus of the eye [67,68,97,105,135,150,157,158,159]. According to Johnson, 1901 [24], this eyelid was also present in other representatives of the Ursidae family (American black bear, Sloth bear, Sun bear). Colitz et al., 2012 [140], Gulland et al., 2018 [138], and Kastelein et al., 1993 [76], reported that in the pinnipeds, the third eyelid was present, and it ran obliquely from rostral-dorsal to ventral-caudal between the eyelids. According to Johnson, 1901 [24], in the seal and sea lion, the third eyelid is well developed. This third eyelid can be depressed by one of the smooth muscles of Müller [76], in contrast to the terrestrial mammals where the movement of the third eyelid is called passive traffic [57]. In the case of Mustelidae in the sea otter, the third eyelid is half-transparent, which is related to the adaptation of these animals to an aquatic residence [160]. In kinkajou, the third eyelid is well developed and forms the conjunctiva a sort of loose bag which contracts over the cornea, while in the raccoon it is vestigial [24]. Morphometric analysis showed that the third eyelid was longer in the South African painted dogs and mongrel mesocephalic domestic dogs than in the subject Asiatic black bear and crab-eating fox [67,68]. The histological study in the examined Asiatic black bear, as well as domestic and wild Canidae, revealed that the cartilage of the third eyelid was surrounded by a thick layer of collagen and elastic fibers and consisted of hyaline tissue [67,68,95,135]. Our research showed the presence of conjunctival lymphoid follicles of the third eyelid, as well as in the crab-eating fox, domestic dogs and South African painted dogs [67,68,135]. The presence of lymphoid follicles in the third eyelid in the Canidae [105] is a physiological condition, and we suppose that in the case of Ursidae, it may be similar because they are part of the immune system responding to viral infections (Canine herpesvirus-1 (CHV-1)) causing follicular inflammation of the third eyelid as well as the presence of foreign bodies in the conjunctival sac, and by antigens present in the dog’s environment, such as dust or pollen [161]. Our analysis did not reveal the presence of the deep gland of the third eyelid (commonly known as the Harderian gland) in the Asiatic black bear similar to its Canidae, but according to Paule, 1957 [162], these glands among the Carnivore investigations are present only in the ranch mink and Eastern raccoon (*Procyon lotor lotor*) and is a small orbital gland with mixed nature. Research conducted by Kastelein et al., 1993 [76], on the walrus also showed of the Harderian gland which the authors call glandular lacrimal accessory and produces an oily mucus and lies rostroventral to the eyeball. Additionally, the first description of the Harderian gland in this species was presented by Owen in 1853 [163] and Pütter in 1903 [164], where it was described as a small gland that produces a mucus-like secretion. As reported by Kastelein et al., 1993 [76], this gland is to prevent the cornea from drying out, and protects the eye from particles and chemicals underwater and also infections.

### 4.5. The Lacrimal Gland

In our study, the lacrimal gland of the Asiatic black bear was located in the dorsal-lateral corner of the eye, between the dorsal and lateral straight muscles inside the periorbital, similarly to the representatives of the family Canidae (*Canis lupus familiaris*, *Cerdocyon thous or Lycaon pictus pictus*) [67,68,151,152,165,166,167]. However, in the case of the sea lion, this gland is also dorsotemporal beneath the superior eyelid but is referred to as temporal ocular gland; it is a typical main lacrimal-like gland, which appears to be lacrimal in nature and therefore is likely to be involved in the principal formation of the aqueous component of the tear [139,156]. As reported by Kelleher Davis et al., 2013 [139], this gland, like the medial ocular gland in the sea lion, has a very similar structure and may be analogous to the aqueous-producing gland of terrestrial mammals. However, the opposite is true in the Cetacea, where, according to Tarpley and Ridgway, 1991 [153], the typical lacrimal gland present in terrestrial mammals is absent, but is on a component of the orbital gland which produces an oil secretion, creating a precorneal tear film. The lacrimal gland had a triangular shape on the Asiatic black bear and as reported by El-naseery et al., 2016 [165], Park et al., 2016 [151], and Zwingenberger et al., 2014 [168], in the case of domestic dogs, the shape of the gland may be different, even about the breed of the dog. One can ask whether there will be differences in the shape of the lacrimal gland in the subfamily Tremarctinae, Ursinae and Ailuropodinae, also taking into account the habitat of these animals, but due to the lack of reports related to the morphology of this gland, this question is, so far, purely hypothetical, presenting a wide field for research on the organ of vision in different mammals. Morphometric analysis showed that these glands in the Asiatic black bear were very small, for example, compared to the tear gland in a South African painted dog, which was a large gland [68] but of similar dimensions in the crab-eating fox [67,105,159,160,161]. Saito et al., 2004 [169], and El-naseery et al., 2016 [165], showed in studies conducted on various domestic dogs, including purebred dogs and mongrel dogs, that the sex, the breed and also the type of skull may influence differences in the morphometry of the lacrimal gland. Histological and histochemical studies have shown that this gland had a multilobar acinar branched complex structure with muco-serous character, in contrast to the studies performed on Canidae and Pinnipedia, where it has a tubuloacinar structure and produces a serum-like secretion in Canidae [67,68,105,138,165,166] and in the sea lion these glands potentially recreate an aqueous component of the precorneal tear film. Our research showed, as in the case of the lacrimal gland in the elderly female of the South African painted dog [68], the presence of a single lymphoid follicle and numerous plasma cells. We suppose that the presence of lymphatic system structures is probably related to the age of the studied specimens, but also inflammatory processes within the lacrimal gland. According to Paszta et al., 2021 [68], in the case of South African painted dog females, the medical-veterinary interview and the post-mortem findings did not reveal any visual changes indicating an ongoing inflammatory process within the orbit. In our case, apart from the influence of age on the presence of the lymphoid follicle in the Asiatic black bear, not only in the lacrimal gland but also on the presence of numerous lymphocyte clusters around the main ducts in the superficial gland of the third eyelid, as we suppose, it could have an outflow of inflammation in the area of the maxillary alveolar arch.

## 5. Conclusions

A detailed characterization of the structure of the orbit, eyeball, eyelids and glands of the eyeball of the Asiatic black bear has not been studied so far. In the structure of the above-mentioned elements, the presence of features typical of the Asiatic black bear but also common between the Ursidae and Canidae families was found, such as:

- The eyeball was a spherical shape, while the periorbita was funnel/conical-shaped and the orbit was an open type;
- The cornea in Asiatic black bear did not include Bowman’s membrane;
- The tapetum lucidum was cellulosum, like other Canidae;
- The individual orbital openings were arranged in a manner typical for Asian black bears (knowledge of their exact location is associated with properly performed local anesthesia, if necessary);
- In the anterior palpebral margin of the upper eyelid, delicate and short eyelashes were observed, while in the lower eyelids they were absent, just like in Canidae;
- The presence of lymphoid follicles, diffuse lymphocytes and high endothelial venules (HEV) within the eyelids as well as in Canidae and from 7–10 conjunctival folds with numerous goblet cells characteristic only to Asiatic black bear;
- The marginal part of the third eyelids was very pigmented and was thick. The third eyelid was T-shaped and present were lymphoid cells that formed the subepithelial conjunctival lymphoid follicle (CALT) characterized to Canidae and Asiatic black bear;
- The superficial gland of the third eyelid was a multilobar vesicular branched complex with seromucous nature and numerous lymphoid cells formed lymph nodes concentrated around the main ducts;
- The lacrimal gland was very small and had a multilobar acinar branched complex structure, producing a muco-serous discharge, a small single lymphatic papule, and numerous plasma cells located in the glandular interstitium.

The current study not only broadens the knowledge of anatomy and comparative anatomy, but it can also be useful especially for veterinarians specializing in working with exotic animals and for veterinary ophthalmologists and oncologists. Nevertheless, considering the small number of animals (*n* = 1), these results should also be considered as preliminary analyses for subsequent anatomical studies in this direction, also with the use of other research methods.

## Figures and Tables

**Table 1:** Histochemical analysis of the *Ursus thibetanus* upper and lower eyelids, superficial gland of the third eyelid, third eyelid and lacrimal gland.

Examined Structure		PAS	AB pH 1.0	AB pH 2.5	AB pH 2.5/PAS	HDI
upper and lower eyelids	tarsal glands	−	−	−	−	−
	sebaceous glands	−	−	−	−	−
	ciliary glands	−/+	−	−/+	−/+ (blue color)	++
	goblet cells	+++	+++	+++	+++ (magenta color)	+++
superficial gland of the third eyelid	serous acini	−/+	−	−/+	+ (blue color)	−
	mucous acini	++	−	−/+	++ (magenta color)	++
third eyelid	goblet cells in the palpebral and bulbar conjunctiva	+++	++/+++	+++	+++ (magenta color)	+++
lacrimal gland	serous acini	+	−	−	++ (magenta color)	−
	mucous acini	++	−	++	++ (blue color)	+

## Data Availability

Not applicable.

## Supplementary Materials

The following supporting information can be downloaded at: https://www.mdpi.com/article/10.3390/ani12070801/s1, Table S1: Review of the morphological analysis of orbital region, eye tunics, eyelids and orbital glands of the Caniformia suborder [23,24,25,26,28,29,30,31,66,67,68,69,72,75,76,83,84,85,86,87,89,91,92,96,97,103,104,106,107,111,113,115,116,117,118,119,121,122,127,130,131,132,135,138,139,143,150,151,152,158,163,164,165,166,167,168,170,171,172,173,174,175,176,177,178,179,180,181,182,183,184,185,186,187,188].
