# Supplementary material for: Morphology and Histology of the Orbital Region and Eye of the Asiatic Black Bear (Ursus thibetanus)—Similarities and Differences within the Caniformia Suborder
Source: Animals (Basel). 2022 Mar 22;12(7):801. doi: 10.3390/ani12070801 (PMC8997068; doi:10.3390/ani12070801)
Supplement: Supplementary file 1 [file animals-12-00801-s001.zip › animals-1623031-supplementary.pdf]

Table S1

Review of the morphological analysis of orbital region, eye tunics, eyelids and orbital glands of the Caniformia suborder.

Infraorder	*Parvordo*	Family	Species	Skull with orbital region	Eye tunics	Upper and lower eyelids	Superficial gland of the third eyelid with third eyelid	Deep gland of the third eyelid	Lacrimal gland	Reference
			Canis familiaris	+	+	+	+	A	+	[24,84,85,86,107,111,113,135,143,150,151, 152,158,165-168,170-174]
		Canidae	*Cerdocyon thous*	+	+ AED, CD TeED	+	+		+	[25,67,87,97,175]
			Chrysocyon brachyurus		+	+	+			[97]
Canoidea			*Canis lupus*	+	+ cornea AED, CD TeED					
			Canis dingo		+					[25,66,84,86]
			Vulpes corsac		+ cornea					[84,86]

			Vulpes vulpes		+ lens pupil retina AED, CD,TeEd					[84,86] [25,66,69,96,115]
			Vuples lagopus		+ retina AED, CD TeED					[25,115]
			Canis lupus		+ AED					[66]
			Canis lupus lupus		+ lens pupil					[96]
			Canis latrans	+	+					[175]
			Canis rufus	+	+					
			Lycaon pictus pictus	+	+	+	+	A	+	[68]
			Lycalopex culpaeus	+						[176]
			Vulpes lagopus	+						[127]
			Nyctereutes procyonoides	+						[130]
			Urocyon cinereoargenteus		+ lens AED, CD TeED					[25,66,177]
			Speothos venaticus		+ AED, CD TeED					[25]

			Alopex lagopus		+<br>*tapetum*					[171]
			Ursus arctos horribilis	+	+					[26,178]
			Melursus ursinus	+	+<br>AED, CD<br>TeED<br>*tapetum*					[25,32,33,178]
			Ursus arctos	+	+<br>AED, CD<br>TeED					[25,28,29,30,31,66]
	Ursida	Ursidae	*Ursus americanus*		+<br>retina<br>AED, CD<br>TeED					[23,25,66]
Arctoidea			*Ursus maritimus*		+<br>retina					
			Selenarctos thibetanus		+<br>AED,<br>CD,<br>TeED					[178]<br>[25]
			Ursus malayanus		+<br>central<br>area					[24]
			Callorhinus ursinus		+<br>retina					[179]
			Zalophus californianus		+	+	+		+	[91,104,139,180]

	Pinnipedia	Otariidae	*Phoca largha*		+					[181]
			Eumetopias jubatus		+					[24,89]
			Arctocephalus pusillus		+					[89]
				+	+					[75,83,119,121,131,138,182]
		Odobenidae	*Odobenus rosmarus*	+	+			+		[76,89,104,132,163,164]
				+	+			+		[75,83,131]
		Phocidae	*Leptonychotes weddellii*		+	+				[92]
			Halichoerus grypus		+ *tapetum*					[106]
			Pusa caspica		+ retina					[116,118]
			Pusa sibirica		+ retina					[89,116,181]
			Pagophilus groenlandicus		+ retina					[116,117,122]
			Cystophora cristata		+					[103,106,111]
			Phoca vitulina		+					[72,182]

										[24]
			Ommatophoca rossi		+					[133]
			Mirounga angustirostris		+		+			[180]
			Mirounga sp.		+					[104]
				+	+					[75,131,181,182,183]
	Musteloidea	Ailuridae	*Ailuropoda melanoleuca*		+ retina					[100]
			Ailurus fulgens		+ AED, CD, TeED					[25]
		Mephitidae	*Mydaus sp.*		+ AED, CD, TeED					[25]
			Meles meles	+ AED, CD TeED						[25,66,128,130]
			Martes foina	+						[128]
			Lutra lutra	+	+ AED *tapetum*					[24,69,128,129,184]
			Lutra annectens		+ AED, CD TeED					[25]

			Lutra marculicollis		+<br>AED, CD<br>TeED					[25]
		Mustelidae	*Mustela putorius furo*	+	+					[161,184,185,186]
			Mustela putorius	+	+<br>retina,<br>*tapetum*					[24,112,185]
			Enhydra lutris		+<br>retina<br>pupil<br>*tapetum*					[99,105,181]
			Mustela vison		+<br>retina			+		[162,187,188]
			Mustela nivalis		+<br>AED,<br>CD,<br>TeED					[25,69]
			Vormela peregusna		+<br>AED, CD<br>TeED					[25]
			Martes flavigula		+<br>AED, CD<br>TeED					[25]
			Martes martes		+<br>*tapetum*					

										[171]
			Ictonyx striatus		+<br>*tapetum*					[171]
		Procyonidae	*Procyon lotor*		+<br>retina<br>ciliary processes<br>choroid<br>AED,<br>CD,<br>TeED			+		[24,25,66,126,162]
			Procyon cancrivorus		+<br>AED					[69]
			Potos flavus		+<br>retina<br>*tapetum*					[24,126]
			Nasua nasua		+<br>retina<br>*tapetum*					[24,126]
			Bassariscus astutus		+<br>AED, CD<br>TeED					[25]
			Bassaricyon alleni		+					[24]

A – absent, AED – axial eye diameter, CD – corneal diameter, TeED – transverse (equatorial) eye diameter, + presence of the anatomical study of the chosen structure

23. Heyward, J.L.; Reynolds, B.D.; Foster, M.L.; Archibald, K.E.; Stoskopf, M.K.; Mowat, F.M. Retinal cone photoreceptor distribution in the American black bear (*Ursus americanus*). *Anat. Rec.* **2020**, *304*, 1–11.
24. Johnson, G.L. Contributions to the comparative anatomy of the mammalian eye. Chiefly based on ophthalmoscopic examination. *Philos. Trans. R. Soc. Lond. Ser. B Biol. Sci.* **1901**, *194*, 1–82.
25. Kirk, E.C. Eye morphology in cathemeral lemurids and other mammals. *Folia Primatol.* **2006**, *77*, 27–49.
26. Kirbas Dogan, G.; Koral Tasci, S.; Dalga, S.; Ilhan Aksu, S. Anatomical and histological studies on the eye of brown bear (*Ursus arctos horribilis*). *Turk. J. Vet. Anim. Sci.* **2020**, *44*, 871–878.
28. Blendea, A.; Gudea, A.; Irimescu, I.; Crisan, M.; Dezdrobitu, C.; Martonos, C.; Bardas, S.; Damian, A. Studies regarding comparative aspects of the splachnocranium in the brown bear (*Ursus arctros*) and lion (*Panthera leo*). *Bull. Univ. Agric. Sci. Vet. Med. Cluj Napoca Vet. Med.* **2013**, *70*, 7–15.
29. Nezami, B.; Eagdari, S. Allometric growth pattern of skull on brown bear (*Ursus arctos* Linnaeus, 1758) of the Alborz Mountain. *J. Appl. Biol. Sci.* **2014**, *8*, 52–58.
30. Mihaylov, R.; Dimitrov, R.; Raichev, E.; Kostov, D.; Stamatova-Yiovcheva, K.; Zlatanova, D.; Bivolarski, B. Morphometrical features of the head skeleton in brown bear (*Ursus arctos*) in Bulgaria. *Bulg. J. Agric. Sci.* **2013**, *19*, 331–337.
31. Yousefi, M.H. Anatomical study of the Iranian brown bear's skull (*Ursus arctos*); A case report. *Iran. J. Vet. Med.* **2016**, *10*, 237–244.
66. Heard-Booth, A.N.; Kirk, E.C. The influence of maximum running speed on eye size: A test of Leuckart's law in mammals. *Anat. Rec.* **2012**, *295*, 1053–1062.
67. Lantyer-Araujo, N.L.; Nascimento Silva, D.; Estrela-Lima, A.; Muramoto, C.; de Azevedo Libório, F.; da Silva, E.A.; Pontes Oriá, A. Anatomical, histological and computed tomography comparision of the eye and adnexa of crab-eating fox (*Cerdocyon thous*) to domestic dogs. *PLoS ONE* **2019**, *14*, e0224245.
68. Paszta, W.; Klećkowska-Nawrot, J.E.; Goździewska-Harłajczuk, K. Anatomical and morphometric evaluation of the orbit, eye tunics, eyelids and orbital glands of the captive females of the South African painted dog (*Lycaon pictus pictus* Temminck, 1820) (Caniformia: Canidae). *PLoS ONE* **2021**, *16*, e0249368.
69. Kemp, A.D.; Kirk, E.C. Eye size and visual acuity influence vestibular anatomy in mammals. *Anat. Rec.* **2014**, *297*, 781–790.
72. Hughes, A. The topography of vision in mammals of contrasting life style: Comparative optics and retinal organization. In *Handbook of Sensory Physiology VII/5: The Visual System in Vertebrates*; Crescitell, F., Ed.; Springer-Verlag: Berlin, Germany, 1977; pp. 613–756.
75. Davis, R.W. Marine Mammals. In *Sensory Systems, Adaptations for an Aquatic Life*; Springer: Berlin/Heidelberg, Germany, 2019; pp. 177–217.
76. Kastelein, R.A.; Zwypfenning, R.C.V.J.; Spekreijse, H.; Dubbeldam, J.L.; Born, E.W. The Anatomy of the walrus head (*Odobenus rosmarus*). Part 3: The eyes and their function in Walrus ecology. *Aquat. Mamm.* **1993**, *19*, 61–92.
83. Mass, A.M.; Supin, A.Y. Adaptive features of aquatic mammals' eye. *Anat. Rec.* **2007**, *290*, 701–715.
84. Merindano, M.D.; Costa, J.; Canals, M.; Potau, J.M.; Ruano, D. A comparative study of Bowman's layer in some mammals: Relationships with other constituent corneal structures. *Eur. J. Anat.* **2002**, *6*, 133–139.
85. Nautscher, N.; Baurer, A.; Steffl, M.; Amselgruber, W.M. Comparative morphological evaluation of domestic animal cornea. *Vet. Ophthalmol.* **2016**, *19*, 297–304.
86. Merindano, M.D.; Canals, M.; Potau, J.M.; Costa, J.; Ruano, D. Morphomterical features of the corneal epithelium in mammals. *Anat. Histol. Embryol.* **1998**, *27*, 105–110.
87. Renzo, R. Parâmetros oftálmicos em cachorro-do-mato (*Cerdocyon thous*, Linnaeus, 1766). Ph.D. Thesis. Universidade Estadual Paulista–UNESP Faculdade De Ciências Agrárias E Veterinárias Câmpus De Jaboticabal: São Paulo, Brazil, 2015.
89. Mass, A.M.; Supin, A.Y. Eye Optics in Semiaquatic Mammals for Aerial and Aquatic Vision. *Brain Behav. Evol.* **2018**, *92*, 117–124.
91. Miller, S.; Whelan, N.; Hope, K.; Nogueira Marmolejo, M.G.; Knightly, F.; Sutherland-Smith, M.; Rivera, S. Survey of clinical ophthalmic disease in the giant panda (*Ailuropoda Melanoleuca*) among North American Zoological Institutions. *J. Zoo Wildl. Med.* **2019**, *50*, 837–844.
92. Welsch, U.; Ramdohr, S.; Riedelsheimer, B.; Hebel, R.; Eisert, R.; Plötz, J. Microscopic anatomy of the eye of the deep-diving Antarctic Weddell seal (*Leptonychotes weddellii*). *J. Morphol.* **2001**, *248*, 165–174.
96. Malmström, T.; Kröger, R.H.H. Pupil shapes and lens optics in the eyes of terrestrial vertebrates. *J. Exp. Biol.* **2006**, *209*, 18–25.
97. Carvalho, C.M.; Rodarte-Almeida, A.C.V.; Beanes, A.S.; Machado, M.T.S.; Galera, P.D. Ophthalmic contribution to assessing eyes of the two neotropical canids: *Cerdocyon thous* and *Chrysocyon brachyurus*. *Vet. Ophthalmol.* **2020**, *23*, 460–471.

103. Sivak, J.G.; Howland, H.C.; West, J.; Weerheim, J. The eye of the hooded seal *Cystophora cristata*, in air and water. *J. Comp. Physiol. A* **1989**, *165*, 771–777.
104. West, J.A.; Sivak, J.G.; Murphy, C.J.; Kovacs, K.M. A comparative study of the anatomy of the iris and ciliary body in aquatic mammals. *Can. J. Zool.* **2011**, *69*, 2594–2607.
106. Braekevelt, C.R. Fine structure of the tapetum cellulosum of the Grey seal (*Halichoerus grypus*). *Acta Anat.* **1986**, *127*, 81–87.
107. Chijiiwa, T.; Ishibashi, T.; Inomata, H. Histological study of choroidal melanocytes in animals with tapetum lucidum cellulosum. *Graefe's Arch. Clin. Exp. Ophthalmol.* **1990**, *228*, 161–168.
111. Ollivier, F.J.; Samuelson, D.A.; Brooks, D.E.; Lewis, P.A.; Kallberg, M.E.; Komáromy, A.M. Comparative morphology of the tapetum lucidum (among selected species). *Vet. Ophthalmol.* **2004**, *7*, 11–22.
113. Yamaue, Y.; Hosaka, Y.Z.; Uehara, M. Macroscopic and Histological vartiations in the cellular tapetum in dogs. *Anatomy* **2014**, *76*, 1099–1103.
115. Malkemper, E.P.; Peichl, L. Retinal photoreceptor and ganglion cell types and topographies in the red fox (*Vulpes vulpes*) and Arctic fox (*Vulpes lagopus*). *J. Comp. Neurol.* **2018**, *526*, 2078–2098.
116. Mass, A.M. Locatization of the high-resolution area in the ganglion cell layer of the Baikal seal *Pusa sibirica* Gm. 1788. *Dokl. Biol. Sci.* **2016**, *467*, 51–54.
117. Mass, A.M.; Supin, A.Y. Retinal topography of the harp seal *Pagophilus groenlandicus*. *Brain Behav. Evol.* **2003**, *62*, 212–222.
118. Mass, A.M.; Supin, A.Y. Retinal ganglion cell layer of the Caspian seal *Pusa caspica*: Topography and localization of the high-resolution area. *Brain Behav. Evol.* **2010**, *76*, 144–153.
119. Mass, A.M.; Supin, A.Y. Retinal ganglion cell topography and retinal resolution in the Baikal seal (*Pusa sibirica*). *Brain Behav. Evol.* **2016**, *88*, 59–67.
121. Landau, D.; Dawson, W.W. The histology of retinas from the pinnipedia. *Vis. Res.* **1970**, *10*, 691–702.
122. Nagy, A.R.; Ronald, K. A light and electronmicroscopic study of the structure of the retina of the harp seal, *Pagophilus groenlandicus* (Erxleben, 1777). *Rapp. P.-v. Réun. Cons. Int. Explor. Mer.* **1975**, *169*, 92–96.
127. Zuoliang, F. Comparative anatomical studies on the skeleton of artic foxes. *J. Econ. Anim.* **2004**, *8*, 80–84.
130. Hidaka, S.; Matsumoto, M.; Hiji, H.; Ohsako, S.; Nishinakagawa, H. Morphology and morphometry of skulls of Raccoon dogs *Nyctereutes procyonoides* and Badgers *Meles meles*. *J. Vet. Med. Sci.* **1998**, *60*, 161–167.
131. Debey, L.B. Osteological correlates and phylogenetic analysis of deep diving in living and extinct pinnipeds: What good are big eyes? *Mar. Mammal Sci.* **2013**, *29*, 48–83.
132. Kastelein, R.A.; Gerrits, N.M. The anatomy of the walrus head (*Odobenus rosmarus*). Part 1. The skull. *Aquat. Mamm.* **1990**, *16*, 101–119.
135. Constantinescu, G.M.; Moore, C.P. Clinical anatomy of the eyelids for small animal practitioners. *Wien. Tierärztliche Mon.* **1998**, *85*, 229–232.
138. Gulland, F.M.D.; Dierauf, L.A.; Withman, K.L. *CRC Handbook of Marine Mammal Medicine*, 3rd ed.; CRC Press: Boca Raton, FL, USA; Taylor & Francis Inc: London, UK, 2018; pp. 517–536.
139. Kelleher Davis, R.; Doane, M.G.; Knop, E.; Knop, N.; Dubielzig, R.R.; Colitz, C.M.H.; Argüeso, P.; Sullivan, D.A. Characterization of ocular gland morphology and tear composition of pinnipeds. *Vet. Ophthalmol.* **2013**, *16*, 269–275.
143. Moore, C.P.; Wilsman, N.J.; Nordheim, E.V.; Majors, L.J.; Colier, L.L. 1987. Density and distribution of canine conjunctival goblet cells. *Investig. Ophthalmol. Vis. Sci.* **1987**, *28*, 1925–1932.
150. Cazacu, P. Researches Concerning the Morphology of the Nictitating Gland in Dogs. PhD Thesis. IASI: Darmstadt, Germany, 2010.
151. Park, S.A.; Taylor, K.T.; Zwingenberger, A.L.; Toupadakis, C.h.A.; Marfurt, C.F.; Good, K.L.; Murphy, C.J. Gross anatomy and morphometric evaluation of the canine and third eyelid glands. *Vet. Ophthalmol.* **2016**, *19*, 230–236.
152. Cabral, V.P.; Laus, J.L.; Dagli, M.L.Z.; PereiraIvia, G.T.; Talieri, I.C.; Monteiro, E.R.; Mamede, F.V. 2005. Canine lacrimal and third eyelid superficial glands macroscopic and morphometric characteristics. *Cienc. Rural.* **2005**, *35*, 391–397.
158. Goller, T.; Weyrauch, K.D. 1993. The conjunctival epithelium of the dogs. *Ann. Anat.* **1993**, *175*, 127–134.
163. Owen, R. On the anatomy of the Walurus. *Proc. Zool. Soc. Lond.* **1853**, *23*, 103–106.
164. Pütter, A. Die Augen der Wassersaügethiere (in German). *Zool. Jahrb. Jena Gustaf. Fisher Verlag.* **1903**, 17, 97–402.

165. El-naseery, N.I.; El-behery, E.I.; El-Ghazali, H.M.; El-Hady, E. The structural characterization of the lacrimal gland in the adult dog (*Canis familiaris*). *Benha Vet. Miedical J.* **2016**, *31*, 106–116.
166. Martin, C.h.L.; Munnell, J.; Kaswan, R. Normal ultrastructure and histochemical characteristics of the canine lacrimal gland. *Am. J. Vet. Res.* **1988**, *49*, 1566–1572.
167. Shaker, M.M.; Walaa, F.O.A. Anatomical and histological study of the lacrimal gland of the adult male dog (*Canis familiaris*). *Glob. J. Bio-Sci. Biotechnol.* **2016**, *5*, 520–524.
168. Zwingenberger, A.L.; Park, A.A.; Murphy, C.J. Computed tomographic imaging characteristics of the normal canine lacrimal glands. *BMC Vet. Res.* **2014**, *10*, 116.
170. Igado, O.O. Skull typology and morphometric of the Nigerian local dog (*Canis lupus familiaris*). *Niger J. Physiol. Sci.* **2017**, *32*, 153–158.
171. Johnson, G.L. Ophthalmoscopic studies on the eyes of mammals. *Philos. Trans. R. Soc. Lond. Ser. B Biol. Sci.* **1968**, *254*, 207–220.
172. Mowat, F.M.; Petersen-Jones, S.M.; Williamson, H.; Williams, D.L.; Luthert, P.J.; Ali, R.R.; Bainbridge, J.W. Topographical characterization of cone photoreceptors and the area centralis of the canine retina. *Mol. Vis.* **2008**, *14*, 2518–2527.
173. Schmitt, E.; Wallace, S. Shape change and variation in the cranial morphology of wild canids (*Canis lupus, Canis latrans, Canis rufus*) compared to domestic dogs (*Canis familiaris*) using geometric morphometrics. *Int. J. Osteoarcheology* **2014**, *24*, 42–50.
174. Salguero, R.; Johnson, V.; Williams, D.; Hartley, C.; Holmes, M.; Dennis, R.; Herrtage, M. CT dimensions, volume and densities of normal canine eyes. *Vet. Rec.* **2015**, *176*, 386.
175. Renzo, R.; Aldrovani, M.; Crivelaro, R.M.; Thiesen, R.; de Barros Sobrinho, A.A.F.; Balthazar da Silveira, C.P.; Garcia, A.P.; Campos, G.C.S.; Werther, K.; Laus, J.L. The eye of crab-eating fox (*Cerdocyon thous*): Anatomical characteristics and normative values of the selected diagnostic tests, morphometry of corneal tissue, and arrangements of corneal stromal collagen fibres. *J. Zoo Wildl. Med.* **2020**, *51*, 280–289.
176. Segura, V.; Prevosti, F. A quantitative approach of the cranial ontogeny of *Lycalopex culpaeus* (Carnivora: *Canidae*). *Zoomorphology* **2012**, *131*, 79–92.
177. Rexford, D. The lens as an indicator of age in the gray fox. *J. Mammology* **1961**, *42*, 109–111.
178. Peichl, L.; Dubielzig, R.R.; Kubber-Heiss, A.; Schubert, C.; Ahnelt, P.K. Retinal cone types in brown bears and the polar bear indicate dichromatic color vision (abstract). *Investig. Ophthalmol. Vis. Sci.* **2005**, *46*, 4539.
179. Mass, A.M.; Supin, A.Y. Peak density, size and regional distribution of ganglion cells in the retina of the fur seal *Callorhinus ursinus*. *Brain Behav. Evol.* **1992**, *39*, 69–76.
180. Turner, E.C.; Sawyer, E.K.; Kaas, J.H. Optic nerve, superior colliculus, visual thalamus, and primary visual cortex of the northern elephant seal (*Mirounga angustirostris*) and California sea lion (*Zalophus californianus*). *J. Comp. Neurol.* **2017**, *525*, 2109–2132.
181. Ninomiya, H.; Imamura, E.; Inomata, T. Comparative anatomy of the ophthalmic rete and its relationship to ocular blood flow in three of marine mammal. *Vet. Ophthalmol.* **2014**, *17*, 100–105.
182. Levenson, D.H.; Schusterman, R.J. Dark adaptation and visual sensitivity in shallow and deep diving pinnipeds. *Mar. Mamm. Sci.* **1999**, *15*, 1303–1313.
183. Hanke, F.D.; Hanke, W.; Scholtyssek, C.; Dehnhardt, G. Basic mechanisms in pinniped vision. *Exp. Brain Res.* **2009**, *199*, 299–311.
184. Ye, Y.; Doung, T.A.D.; Saito, K.; Shinmyo, Y.; Ichikawa, Y.; Higashide, T.; Kagami, K.; Fujiwara, H.; Sugiyama, K.; Kawasaki, H. Visualization of the retina in intact eyes of mice and ferrets using a tissue clearing method. *Transl. Vis. Sci. Technol.* **2020**, *9*, 1–9.
185. He, T.; Friede, H.; Kiliardis, S. Macroscopic and roentgenographic anatomy of the skull of the ferret (*Mustela putorius furo*). *Laboratoty Anim.* **2002**, *36*, 86–96.
186. Hernandez-Guerra, A.M.; Rodilla, V.; Lopez-Murcia, M.M. Ocular biometry in the adult anesthetized ferret (*Mustela putorius furo*). *Vet. Ophthalmol.* **2007**, *10*, 50–52.
187. Tjälve, H.; Frank, A. Tapetum lucidum in the pigmented and albino ferret. *Exp. Eye Res.* **1984**, *38*, 341–351.
188. Braekevelt, C.R. Fine structure of the retinal epthelium and tapetum lucidum of the ranch mink *Mustela vision*. *Acta Anat.* **1989**, *135*, 296–302.
